# Direct Compaction Drug Product Process Modeling

**DOI:** 10.1208/s12249-021-02206-4

**Published:** 2022-01-31

**Authors:** Alexander Russell, John Strong, Sean Garner, William Ketterhagen, Michelle Long, Maxx Capece

**Affiliations:** 1 Operations Science & Technology, AbbVie, 67061 Ludwigshafen, Germany; 2 R&D Drug Product Development, AbbVie, North Chicago, Illinois 60064 USA; 3 Operations Science & Technology, AbbVie, North Chicago, Illinois 60064 USA

**Keywords:** direct compaction, process modeling, drug product, process engineering, pharmaceutical technology

## Abstract

Most challenges during the development of solid dosage forms are related to the impact of any variations in raw material properties, batch size, or equipment scales on the product quality and the control of the manufacturing process. With the ever pertinent restrictions on time and resource availability versus heightened expectations to develop, optimize, and troubleshoot manufacturing processes, targeted and robust science-based process modeling platforms are essential. This review focuses on the modeling of unit operations and practices involved in batch manufacturing of solid dosage forms by direct compaction. An effort is made to highlight the key advances in the past five years, and to propose potentially beneficial future study directions.

## INTRODUCTION

The standard dosage form for oral pharmaceutical products continues to be the tablet as evidenced by approximately 60% of the oral drug products approved by the FDA so far in 2021 [1]. The design allows for convenient unit dosing in a physically and chemically stable form that provides robustness to shipping and handling processes. The considerations required in the design and manufacture of tablets provide a rich field for exploration and innovation for pharmaceutical scientists, material scientists, chemical and mechanical engineers. Each of the tablet materials brings with it unique properties that affect handling and processing during manufacturing. In addition, material properties may prompt the selection of specific manufacturing unit operations and constrain operating conditions. Beyond the active pharmaceutical ingredient(s), the formulation will contain pharmaceutically inactive additives that increase the bulk of the product and function as diluents to improve manufacturability, improve compressibility, act as binders to maintain integrity, contribute enhanced disintegration and dispersion to facilitate gut dissolution, or conversely contribute as controlled release agents to retard or extend drug release [2]. Often the final presentation includes coating agents for cosmetic and/or functional purposes. Further, the selection of physical size and shape of the final tablet can introduce complex mechanical interactions and inhomogeneous force distributions that influence the force and duration of compression steps necessary for tablet formation. These same mechanical and material properties lead to surfaces with varying physical-chemical properties and physical features that will affect the adhesion and efficiency of applied coatings. The size, shape, and markings on the tablet will vary to enable the unique identification of the dosage form as well as a variety of considerations including patient ability to swallow, need for dosing flexibility, and distinctive features to minimize dosing errors or represent a company’s preference for branding.

**Fig. 1:**
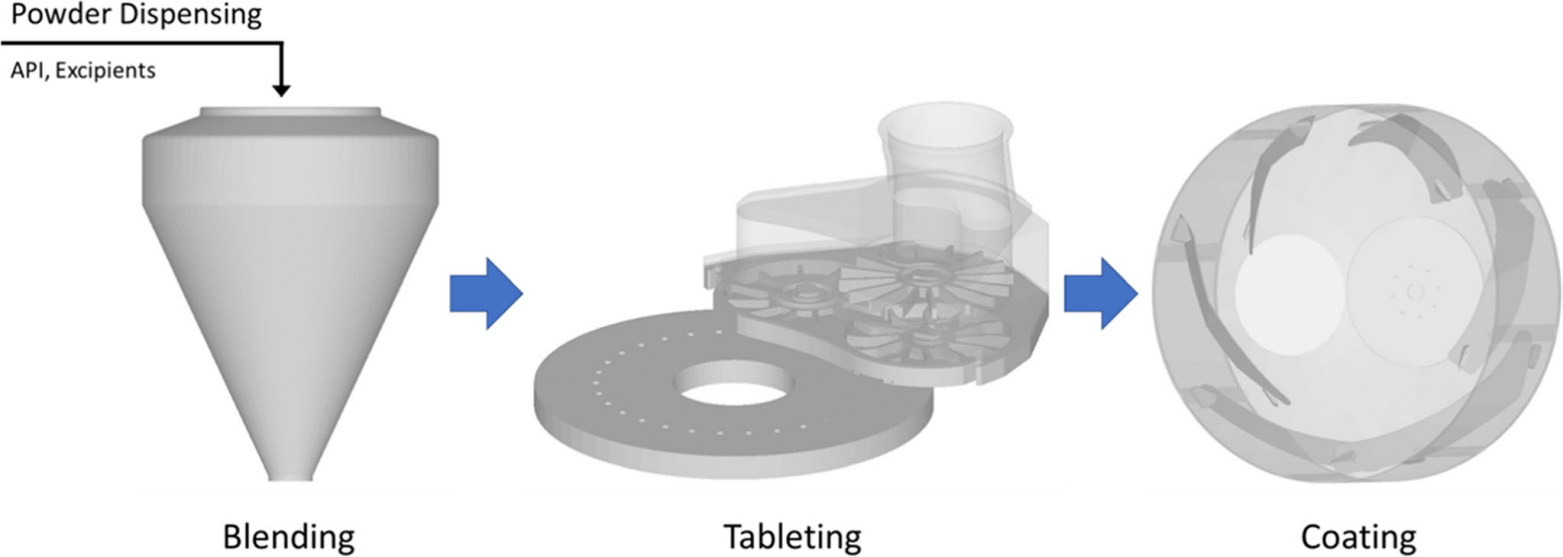
Order of direct compaction unit operations

As part of the continuous effort to reduce risk in manufacturing with consideration of the variables described above, the pharmaceutical industry is seeking to increase the design and development of formulations suitable for robust direct compaction processes. The basic sequence of unit operations for a direct compaction process resulting in a coated tablet is depicted in Figure 1.

The straightforward nature of the unit operations involved brings the opportunity to implement continuous processing for large volume products. However, the apparent simplicity belies the complexity invoked by relying on the intrinsic and extrinsic properties of the formulation materials throughout manufacture. This fact also leads process modelers away from continuum models to discrete particle models to improve alignment with observed phenomena.

For the process engineer, direct compaction relies on the appropriate selection of formulation ingredients and potentially some upfront work on particle engineering of API due to the elimination of a granulation step historically included in tablet manufacture [3]. Wet or dry granulation unit operations are particle engineering steps that create excipient/API granules with larger aggregate particulates that improve flowability, compressibility, and importantly, content uniformity especially for drug products with low dosage strengths. Without a granulation step, direct compaction processes are strongly reliant on control of powder flow since there is greater susceptibility to segregation of dissimilar materials during unit operations. Further, tableting performance is more reliant on individual material physical properties and adhesive interaction under compression. Unit operation models developed for direct compaction incorporate the same phenomena of particle flow, plastic deformation, and adhesion on compression found in process models that include granulation. However, the individual contributions of the formulation materials require the models to maintain the unique contributions of each material throughout the process, thus reducing the opportunity to create simplifying pseudo homogeneous material assumptions available after granulation steps or within process corrections of inhomogeneity. While models and simulation approaches continue to evolve with ever-increasing capabilities to capture the physical and thermodynamic interactions resulting in practical outcomes, all models will have inherent assumptions. Depending on the similarity of the materials, simplifying assumptions can be applied to allow modeling as if dealing with, for example, a uniform bulk material of homogeneous spherical particles of narrow size distribution. However, the realities of pharmaceutical formulations require models to incorporate variations in particle size, size distribution, shape, and density among other properties that must be refined and validated through supporting experimentation. Examples of these influences and approaches for reflecting the impact in modeling are discussed within this review.

Well-designed process models are predictive and demonstrate knowledge and understanding. For any modeler, the approach selected will depend on the intended use of the model. Early in development, the use of first principles computationally intensive models, approaches such as Computational Fluid Dynamics (CFM) and Discrete Element Method (DEM), contributes to understanding the potential importance of various material properties. At this stage, experimentation and modeling work hand in hand, where the model may suggest behavior, and the experimental work will either confirm or suggest a missing parameter in the model. As the product development program moves further in development, there is a need for models that are less computationally intensive and can provide guidance and confirm experiment meets expectations more immediately. Finally, the models can be tuned to the process in a way to provide digital twins or be used for process monitoring and control feedback which is the expectation and need especially for continuous manufacturing. As mentioned previously, direct compaction processes lend themselves to continuous manufacturing. Models used in understanding during process development can evolve into or help inform the predictive models needed for process monitoring and control of a continuous process. The transition in modeling approach is necessary as the models used during development are frequently computationally intensive and time-consuming whereas process models for control need to respond in real-time[4, 5].

This review will explore the models available for the development of the unit operations involved in batch manufacture of direct compaction drug products. It is written from the perspective of process engineers enabling the manufacture of a formulation designed to fit for purpose. In practice, however, the discussion of product design intent and selection of materials is best conducted with integrated teams to understand the control strategy needed in the manufacturing process to realize the critical quality attributes of the final dosage form. Manufacturing process models can assist in the selection of materials by illustrating the impact of material properties such as particle size distributions or plasticity while allowing formulation scientists to select materials that will fulfill intended purposes such as promoting disintegration or modifying drug release. In addition, the application of process models can contribute to the optimization of manufacturing equipment as will be further discussed below. Integration of modeling throughout development enables material sparing strategies while exploring the process design space. This combined effort leads to scientifically robust designs and process understanding expected for developing risk-based decisions for process and product control supporting the concept of Quality by Design (QbD) as described in ICH Q8 [6–8].

## MODELING OF BATCH PROCESSES

For the unit operations covered in this review, the modeling approaches will mainly focus on thermodynamic and physics-based models. In several cases, the models described can be applied generally and are not specific to direct compaction. The review begins with a discussion of power flow representing charging of materials and conveyance of blends, then continues into blending operations. These sections are followed by reviews of tableting and coating processes. Often, for direct compaction formulations, the models are considered at the individual particle level. Thus, material physical properties such as particle size and size distribution, shape, surface energy, and mechanical properties are commonly invoked. The relative importance of these parameters depends on the unit operation involved. Particle size, distribution, and shape affect granular flow and material segregation. Surface energy and mechanical moduli reflecting plasticity can affect material flow and adhesion during compaction. After tableting, for the subsequent operations such as coating, the formed tablet is now the particle.

In these discussions, there are similar modeling approaches applied; however, the reader is directed to other sources for detailed explanation of the methods as contextually cited in the sections below. All models will require simplifying assumptions to be computationally tractable and responsive to the time frame in which results are expected. While many are supported by commercial packages, development and application of the models often require people with dedicated expertise or access to external subject matter experts. For convenience, the following brief descriptions of platform modeling techniques are provided.

### Computational Fluid Dynamics (CFD)

Computational Fluid Dynamics (CFD) uses a continuum assumption to numerically simulate flow using constitutive equations and the concepts of conserving mass, momentum, and energy. Useful for the flow of material except as approaching the close-packed limit where interparticle interactions dominate.

### Finite Element Method (FEM)

Numerical approach to solve a partial differential equation. It is generally employed by studying the domain parsed into finite elements connected by nodes (referred to as a mesh). The approach develops equations for each element and then assembles them for the entire domain and solves it. The approach originally developed for soil mechanics is highly useful for studying stress-strain patterns in powder mechanics.

### Discrete Element Method (DEM)

Numerical simulation technique that uses Newton’s second law to follow the motion of individual particles and their interactions with other particles. These interactions can be hard-sphere (elastic and instantaneous) or soft sphere which enables simulation of extended contact as could be caused by the presence of large solids fractions and compounded by the effect of friction or plastic deformation. Because this method is tracking individual particles, it is often limited to a finite set of particles to reduce the computational burden.

### Population Balance Modeling (PBM)

Mathematical modeling approach that can describe how a particle population evolves in one or more specific properties over time. The model quantifies the rate of change of the number density function of the tracked property — describing the mean system behavior of the particle population from the analysis of single-particle behavior. This is done by computing the difference between the number of particles that form and correspond to a certain value of the property and the number of particles depleting from those exhibiting that value within the same time. Given the nature of this approach, PBM is often used to study rate-governed processes, e.g., crystallization, granulation, and milling. The higher the number of properties being tracked, the higher will be the dimension of the partial differential equation describing the process. Generally, these equations are solved numerically as analytical results may be available for only simplified cases.

### First-Principle Approaches

These models are often derived from the constitutive equations for mass and energy balance. There can be equipment-specific parameters that are derived from experimental measurements. The expressions are useful for unit operations that are well understood and are not significantly influenced by small-scale localized phenomena or individual particulate behavior. Useful scaling methods are often developed using nondimensional numbers defined from parameters of the process or material representing the balance of forces involved in the unit operation or phenomena of interest. Unlike the computational methods, these models will often provide analytic solutions that are more generally accessible and can be used to predict process behavior once the expressions have been fit to process data to obtain process constants.

### Empirical Methods

Not all modeling is based on applying first principles or direct representations of operative phenomena. Empirical models derived from data generated by the process or unit operation of interest can capture the influence of specific factors such as environmental, material, or process parameters. Linear regressions, statistical design of experiments, and multivariate approaches such as principal component analysis are common examples. These models are useful for performance characterization and yield opportunities for process monitoring but are less informative when seeking process understanding that can contribute to a knowledgebase transferrable to other situations.

### Powder Feeding, Discharge, and Flow

#### Introduction

The range of flow behaviors that powders exhibit are diverse, resulting from constituent particle properties, particle-particle interactions, and the local stress condition. These three factors are interdependent, whereby changes in one profoundly impact the others and the consequent momentum transport, altering the flow behavior. Therefore, powders alternate between exhibiting fluid-like and solid-like behaviors, mandating the need to understand the limits of a process where the flow will be reliable and consistent. Since the challenge to control the powder to flow consistently and smoothly throughout the process is complex, research has mostly focused on cases specific to formulations and equipment.

Starting from the early 1980s until the introduction of computational techniques, contributions were elucidations of physics-based models and their use to evaluate specific experimental measurements. Since the accepted application of computational modeling in studying pharmaceutical powder mechanics by the late 1990s, the discrete element method (DEM), alone and in combination with computational fluid dynamics (CFD), has helped quantify powder behavior in diverse geometries and conditions. The first review on pharmaceutical process modeling [9] gives a clear and holistic description of the fundamental physical meanings of the various discrete models available to study powder flows. The recent book chapter by am Ende et al. [10] reiterates the fundamental working principles of DEM and DEM-CFD and lays down the operational procedure and limitations of the approaches.

In a typical direct compression tablet manufacturing process, the flowability of powders needs to be considered during filling into a container for blending, feeding/discharging the blend into the compression equipment hopper, and blend flow into the die for compression. At all times, the key objective is to retain the degree of mixedness after blending. This section presents recent advances in efforts done to improve powder feeding, discharge, and flow as well as to minimize potential segregation by sifting or entrainment, serving as a prelude to the subsequent sections discussing blending and compression.

#### Sub-processes

##### Charging of Formulation Powders

Filling or charging components into an intermediate bulk container (IBC) or blender is generally done without issue. For blends with low drug loadings, or when the components exhibit significant differences in particle size and density between each other, it may be beneficial to add the components in alternating small installments to minimize the time required for blending and the effect of losses [11]. However, this is effective only when the components are charged in the direction orthogonal to the axis of rotation.

Of key importance is the powder fill level in the container (blender/IBC). Since each of the materials is not charged simultaneously, the higher the fill level, the lower will be the degree of mixedness to start with, and consequently longer the mixing time needed to achieve homogeneity [12, 13]. The fill level ranges for robust mixing need to be chosen specifically per container geometry, as it would limit the shear stress per unit volume of the material, which along with convection and dispersion drives the mixing process, see [14]. In general, it is accepted that fill levels do not exceed 70% for tumble mixing [15].

##### Blend Discharge from Hoppers/Blenders

Instantaneous initiation of powder discharge from a bin or hopper and maintaining a steady mass flow rate are essential for robust manufacturability. Maintaining blend uniformity during discharge requires that the flow pattern does not systemically induce de-mixing by sifting and/or fluidization. This requires a deeper understanding of the flow trajectories and the interparticle state of cohesion during the flow process.

**Fig. 2:**
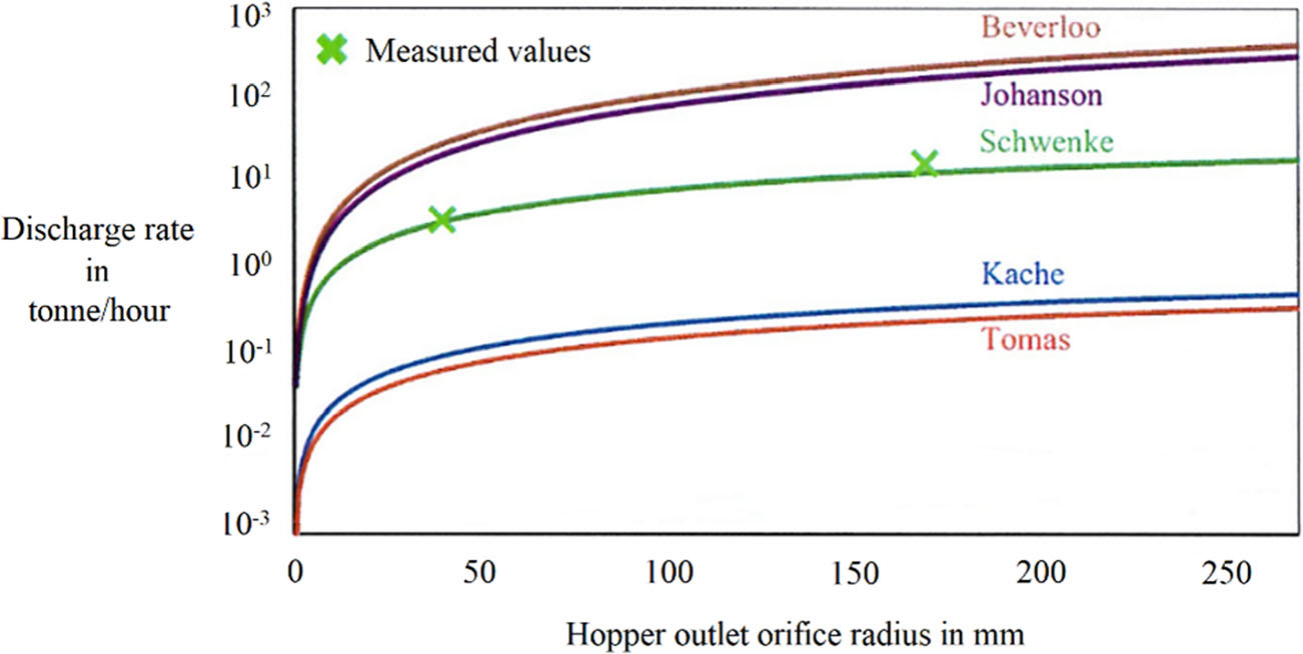
Comparison of the instantaneous discharge rates as a function of hopper outlet radius. Measured data refers to lab-scale experiments using Mikro PVC powder of d_50_ ≈ 4 μm and ff_c_ ≈ 1-2; for compared Models refer to [19–22]. Figure reproduced from [16]

Quantifying the discharge rate may be challenging as the air resistance may significantly impact the force equilibrium in bulk powders, depending on the size, shape, and compressibility of the particle population. In the past couple of decades, there has been progress in understanding this fundamental problem through multiple miniature and computational studies which showed that cohesive powders flow poorly during a purely gravity-driven discharge. Multiple contributions have approached this challenge by using a particle size 100 times larger than the Sauter mean diameter for the calculation of the fluid resistance. Recently Schwenke [16] recalculated the Molerus’s discharge rate [17] by assuming that the interstitial air is not a motionless fluid but moves with a velocity as a function of the flow resistance. The described model appears powerful presenting compelling predictability for four fine powders (d_50_ < 10 μm) with flow function coefficients ranging between 1 and 4. Figure 2 provides an example of one such powder sample. A similar study [18] considered the drag on surrounding particles rather than the discrete particle in itself and presented an empirical approach to predict discharge rates in IBCs based on lab-scale orifice discharge experiments. On one hand, the approach is non-destructive and any number of calibration experiments can be done to predict the linear relationship between the lab scale and commercial scale IBCs. However since the predictability will suffer in accuracy when the drag equilibrium is not consistent, i.e., when the flow is not smooth from a continuum perspective, calibration experiments may be inevitable.

**Fig. 3:**
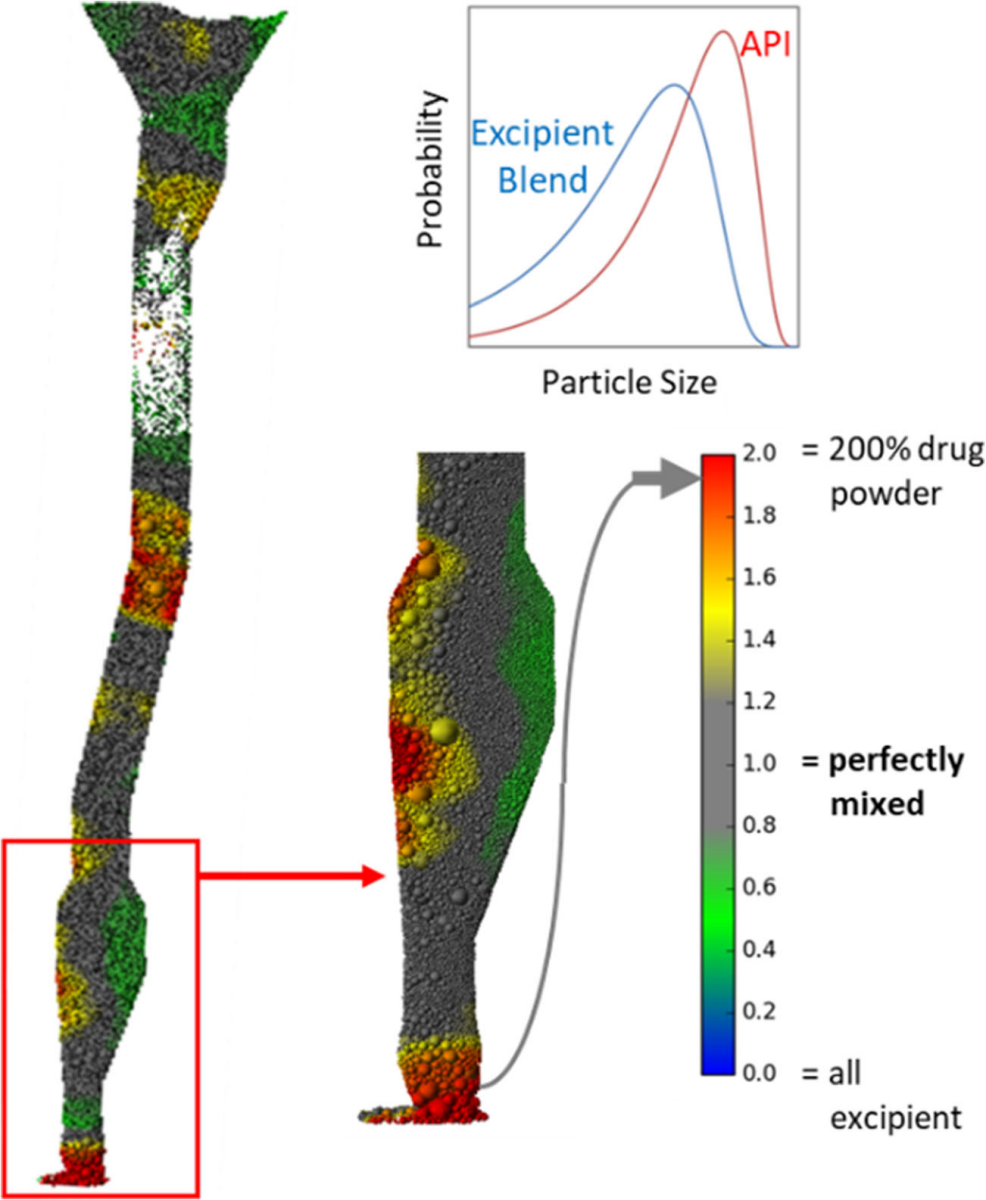
Simulation snapshots of powder transfer system showing API loading for an actual API/excipient particle size distribution demonstrating significant segregation with superpotent tablets at the start of the batch. Figure reproduced from [10]

In general, discharge of blended powder from a bin into the hopper of a tablet press is carried out by maintaining short transfer distances. Such a set-up is in general used to minimize segregation effects by fluidization and by sifting. Ketterhagen et al. [10] evaluated the potential for segregation during free-fall of a blended powder into the hopper of a tablet press through a transfer chute (bent twice slightly), considering different particle size combinations of the API with the rest of the constituents. The simulations demonstrated how dissimilarity in size of the API particles in comparison to the rest of the blend constituents can result in segregation impacting the potency of manufactured tablets throughout the batch — relatively large API can lead to quicker settling by gravity and hence higher potency at the start of the batch, relatively smaller API particles can generate the reverse result in manufacturing with lower potency at the start of the batch (Fig. 3).

##### Powder Feeding to the Press

The flow of powder blend into the die of a compression press is generally controlled by the operating parameters of the compression press — the geometry of the feed frame paddles and the rotation speed and direction of the turret and the feeder-frame paddles. Significant contributions using DEM have provided insight describing the flow patterns of the powders in the feed frame [23].

Mateo-Ortiz et al. [24] studied particle velocities in a lab-scale Manesty Betapress feeder frame and estimated weight-driven segregation per operating speed. A follow-up study [25] for the same feeder frame identified that increasing fill levels between the paddles by modifying operating speeds can minimize percolation segregation. Ketterhagen [26] simulated a lab-scale Korsch XL100 and showed that paddle geometry can impact the residence time distribution of particles in the feeder and could potentially impact the extent of lubrication and changes to the PSD, depending on the material-specific lubrication sensitivity and friability, respectively. Hildebrandt et al. [19, 27] simulated the so-called Fill-O-Matic, a more complex three-stage filling system of the Fette 1200i tablet press. They studied the trajectories of particles of different sizes in each filling stage and showed that larger particles, due to their relatively higher kinetic energy enter the dies first forming the lower layers, and also accumulate at the edges of the paddles. Therefore a percolation segregation mechanism, consistent with findings of Mateo-Ortiz [24, 25], and risks of radial segregation were presented. The turret speed and feeder wheel speed were shown to have the largest effects on tablet quality and should not be independently optimized [28].

Overall, a long residence time in the feeding zone would pose the risk of particle attrition and over lubrication that can impact manufacturability and potential for mechanical defects. On the other hand, a shorter residence time can also pose the risk of segregation downstream from the initial mixing process [26]. Therefore, a certain degree of mixing capability can be beneficial in maintaining content uniformity and minimizing tablet weight variability. Although a robust design space would be dependent on the inter-particle cohesivity of each formulation, the established process insights allow for qualitative remedies. With this feasible modeling methodology, an initial design space estimate for robust processing can be identified at early process design stages when material availability is limited.

#### Modifications to Improve Powder Flow

##### Hopper Design

Modifying angles in hopper outlet design has been a conventional go-to method for very poor flowing powders. In essence, the smaller the opening size and the flatter the angle of the opening, the higher is the content of the powder volume experiencing larger normal stresses. Based on Jenike’s pioneering works [29, 30] designing hoppers with different hopper outlet angles and diameters has been a routine practice, however requiring a careful understanding of the powder properties. A flow chart along with a straightforward step-by-step procedure to solve the complex calculations for hopper wall steepness and outlet dimensions was first presented by Mehos [31]; see also Leung et al.’s [32] risk assessment methodology. Without considerations of air resistance, Hancock [33] highlighted how blends may require hoppers with steeper walls and more smoothly polished surfaces to minimize the risk of erratic flows, sifting segregation, and yield losses.

**Fig. 4:**
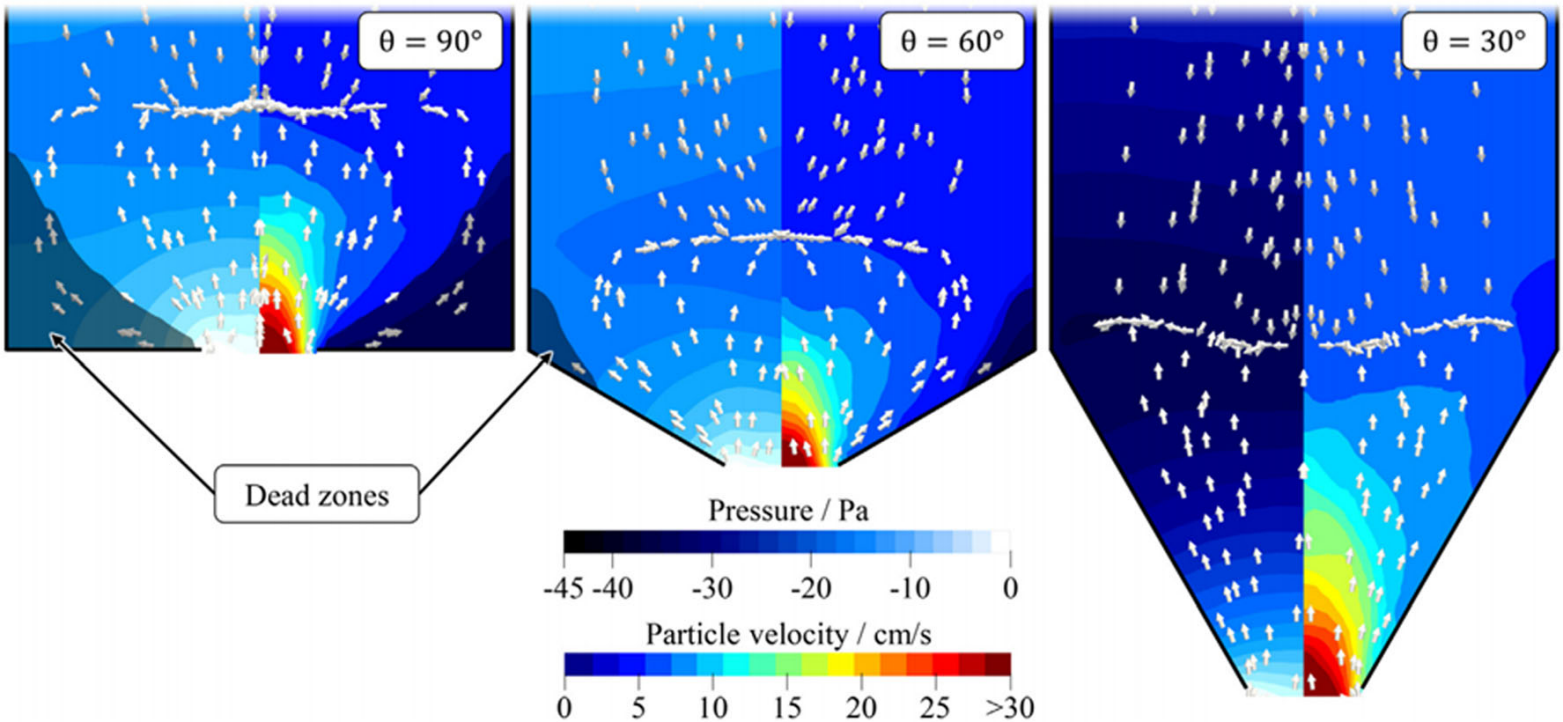
Relative airflow (arrows), air pressure (left half), and absolute particle velocity (right half) at steady state for varying hopper angles from CFD-DEM simulations; dead zones (particle velocity < 4 cm/s) are emphasized [34]

Using DEM-CFD models based on comprehensive material characterization of lactose powders, Hesse et al. [34] showed that the vertical pressure gradient is significantly impacted by the horizontal airflow, and thereby the discharge rates can be different from expectation (Fig. 4). In steep hoppers, strong air impairment can limit one from achieving high discharge rates as per expectation. For wider hopper orifices, the pressure gradient increases due to higher particle velocities and faster relative fluid flow.

##### Hopper Inserts

**Fig. 5:**
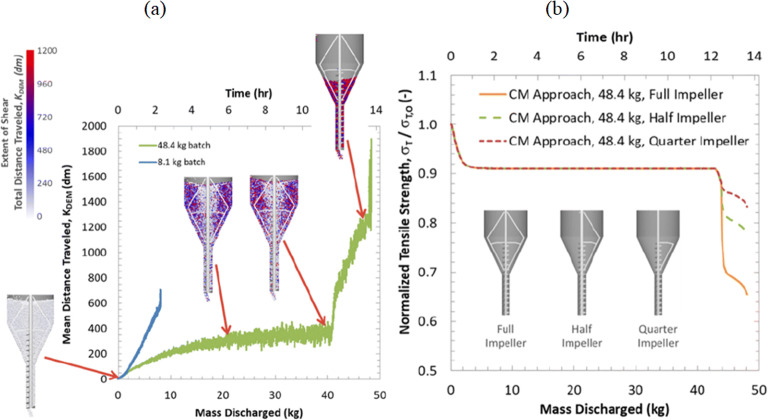
**a** Shear predicted by DEM as the mean distance particles traveled as a function of the discharged mass; **b** Compact tensile strength considering varying impeller sizes. Reproduced from [35]

Another generally accepted approach to improve the discharge of cohesive powders is utilizing different geometries of inserts within the hopper. Ketterhagen et al. [35] discussed using DEM for the effect of an impeller used in the hopper of an encapsulator. Although the formulation used in the study was considered to have a flow function coefficient (ffc) of larger than 10, the simulations were able to identify and distinguish between two regions (Fig. 5a)— a central flow channel and a peripheral agitated stagnant zone. The simulations show that the flow field approaches a steady-state as powder enters the encapsulator hopper and is exposed to minimal shear as it moves through the central flow channel and discharges through the outlet; whereas the material discharging towards the end of the process appears to have spent a longer residence time in the hopper at the annular relatively stagnant zones experiencing higher shear input. The authors also showed that through DEM’s evaluation of the flow profiles and shear energy absorption, the change in tensile strength of thereby made compacts can be predicted to follow three phases: an initial start-up transient high phase, a steady-state phase, and a significantly dropping final phase during hopper emptying (Fig. 5b).

##### Modifying Formulation Surface Properties for Better Flowability

In manufacturing equipment, where material may need to flow through enclosed geometries and engineering adaptations may be undesirable, improving the formulation flowability may be effective to develop or maintain robust manufacturability. Modifying particle surfaces by coating can significantly alter the contact behavior of the single particles [36], and the van der Waal’s potential can be overcome more effectively thus improving flowability [37].

Although surface coating with nanopowders has traditionally been known to aid bulk solids handling efforts, until recently, not much literature has been devoted to demonstrating the benefit and effort of the approach for pharmaceutical cohesive powders. Capece et al. [38] identified that the ffc of cohesive powders correlates to a power-law relationship with the granular Bond number (ratio of the van der Waals interparticle attraction to the particle weight). The authors extended the feasibility of estimating flowability based on fundamental particle properties from monodisperse single-component powders to considering multi-component blends, and different size fractions of the same component. With this fundamental basis, Acetaminophen and Avicel were experimentally demonstrated to flow better when the granular Bond number was decreased by dry coating with coatings of Aerosil R972 [38, 39].

**Fig. 6:**
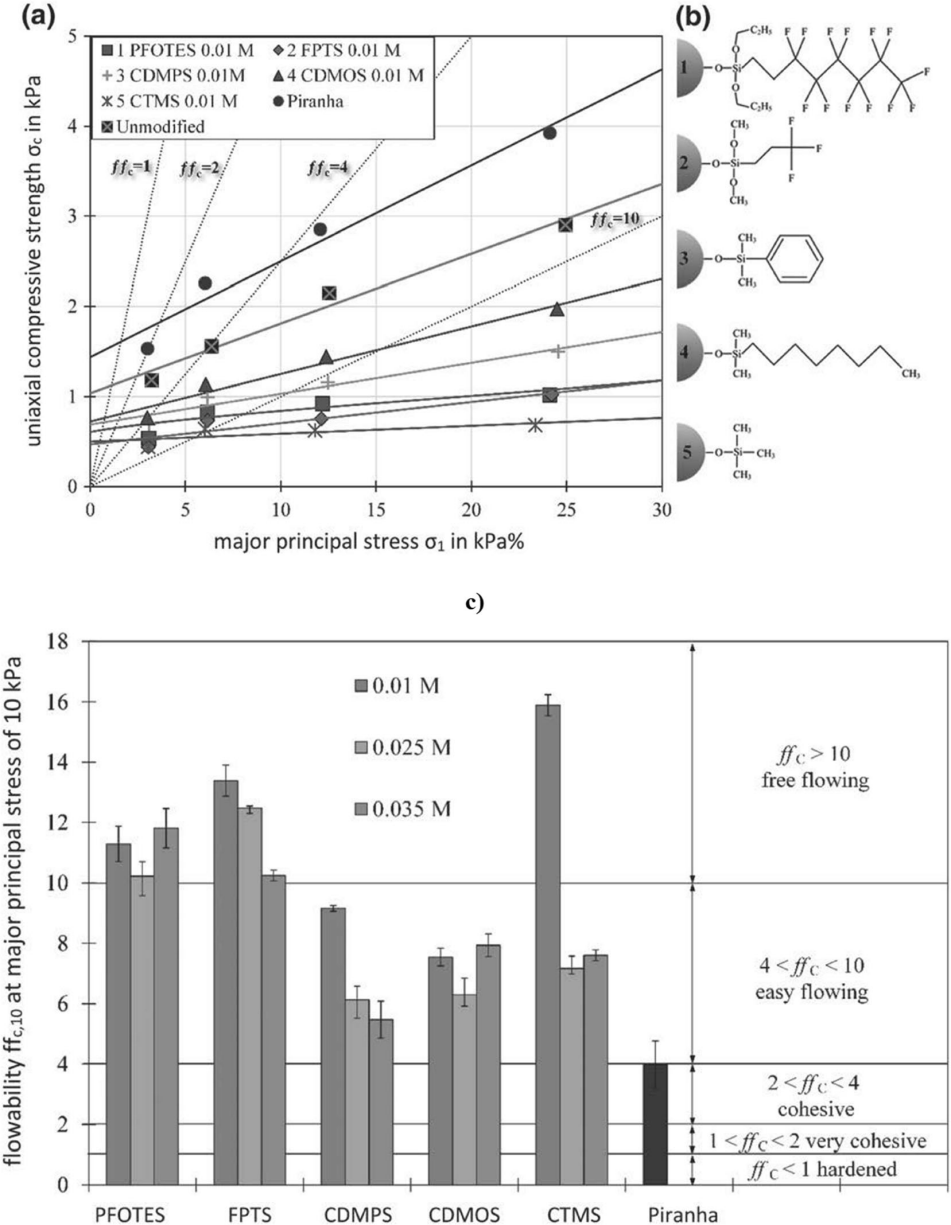
**a** Flow function of silanized glass powder (d_50_ = 8 μm) with a concentration of 0.01 mol/L. **b** Chemical formulas of the used hydrophobic silane coatings —(1) PFOTES: Perfluorooctyltriethoxysilane; (2) FPTS: Trifluoropropyltrimethoxysilane; (3) CDMPS: Chlorodimethylphenylsilane; (4) CDMOS: Chlorodimethyloctylsilane; (5) CTMS: Trimethylchlorosilane; Piranha – peroxymonosulfuric acid. **c** Flow function coefficients of the silanized particles at major principal stress of 10 kPa. Reproduced from [41]

Recently, Escotet-Espinoza et al. [40] demonstrated the effectiveness of blending two APIs with ffc values of 3 and 4 with 1% silica and thereby improving the ffc to 11 and 35 respectively. Towards the same goal, Todorova [41] showed that hydrophobisizing the surfaces of particles can decrease the free surface energy and minimize the effect of capillary forces. Sunkara and Capece [42] arrived at the same conclusion where Aerosil R972 was found to improve flowability better than Aerosil 200 or Cab-O-Sil M5P, owing to its hydrophobic surface and low surface energy. These effects in turn holistically reduce the van der Waals work of adhesion during interparticle contact interactions, thereby improving flowability (Fig. 6). However, the use of the glidant has to be carefully designed such that the interparticle bonding strength is not reduced beyond a lower minimum required for the tablet’s physical integrity. Therefore, coating only the API with the glidant followed by blending with other excipients shows the best improvement in flowability while minimally affecting tabletability [43].

The coverage of glidants on the API particles should be sufficient to reduce adhesive contact of exposed areas and yet maintain sufficient integrity to be retained despite shear forces generated in the process. Although identifying this level of optimum surface alteration appears arduous for specialty particulates, from a methodical perspective, the approach promises an advantage for regular use of general formulation components in known equipment.

#### Advances in Computational Process Modeling Approaches

##### Particle/Assembly Model and Contact Solving Approach

The solving procedure involving the model representing the contact interaction is of deep importance, especially when considering non-spherical particles, bonded single/multi-component agglomerates, and soft materials that are capable of not just a microscopic change at the contact surface but a significant change in their macroscopic shape. A few recent contributions offering advantages in this area are briefly discussed here.

The Material Point Method (MPM) that adds in a continuum perspective in the representation of discrete particles has been known to offer benefits in understanding large deformations. This is generally done by representing each particle as a collection of material points (Lagrangian) and the simulations are carried out with a designed mesh (Eulerian). Although the approach appears beneficial for rapid initial assessments of large domain flows, the accuracy in the dynamics has been known to suffer from over-predictions of particle velocities, since the different contact velocities at the nodes remain unknown. Nezamadai et al. [44] added contact mechanics to nodes to help mitigate the extent to which constitutive laws can be used for the simulation of bulk systems, while Yuan et al. [45] from a fractal dimensions approach considered contact deformations of asperities by merely understanding the asperity geometries and contact forces. Both studies appear beneficial to build-in inhomogeneity in the bulk, while the latter specifically promises benefit in understanding the behavior of powders where particles may contain rough surfaces.

Shen et al. [46] demonstrated the feasibility of using DEM to simulate particle contacts that can lead to adhesion and or detachment by modeling an assembly of primary particles — where a bond material forms after two particles come in contact, or the bond material forms in the gap between two neighboring particles that are not in physical contact (i.e., virtual contact). The first type is commonly referred to as a parallel bond and promises good accuracy to predict mechanical responses of thin bonds, and the second type is commonly referred to as a serial bond which promises good accuracy in predicting the mechanical response of thick bonds. The deformation of the particles and the bonds are modeled through the overlap at the contact which is controlled by the stiffness. Considering cases where liquid bridges or less strong bonds may need to be considered, the contact model proposed by Tsunazawa et al. [47] which supplements the solid contact with capillary forces according to Israelachvili [48] appears beneficial.

##### Constitutive Modeling as Applied to Power Flow

The contact model choice, be it for use in approximating experimental load-displacement or load-time data or for simulating particle dynamics, is essential to accurately represent the underpinning physics. A plethora of models are available as of today for this purpose; Thornton [49] presents an excellent summary of the models proposed until ~ 2014. This sub-section adds on by highlighting key aspects of two models applicable for pharmaceutical processing not covered in Thornton’s compilation.

(i) Elastoplastic adhesive according to Pasha et al. [50]

The model is piecewise linear adapting a linearized representation of the JKR model on Van der Waals attraction [51], and the elastic loading-unloading according to Luding [52], and a linearized plastic loading based on yielding force and deformation according to Thornton [53] and Johnson [54]. The force-displacement f(α) expressions are presented in Eqs. (1-5) in terms of R*, the effective radius of contact curvature, Γ the van der Waals’s attractive distance, k the stiffness; subscripts: e elastic, p plastic, 0 origin, cp irreversible displacement after unloading.

1 $$ \mathrm{Adhesive}\ \mathrm{attraction}\kern0.75em \mathrm{f}=-4/3\cdotp \uppi \cdotp \mathrm{R}\ast \cdotp \Gamma $$

2 $$ \mathrm{Elastic}\ \mathrm{loading}-\mathrm{unloading}\kern0.5em \mathrm{f}=\left({\mathrm{k}}_{\mathrm{e}}\cdotp \upalpha \right)+\left(4/3\cdotp \uppi \cdotp \mathrm{R}\ast \cdotp \Gamma \right) $$

3 $$ \mathrm{Elastoplastic}\ \mathrm{loading}\kern0.75em \mathrm{f}={\mathrm{k}}_{\mathrm{p}}\left(\upalpha -{\upalpha}_{0\mathrm{p}}\right) $$

4 $$ \mathrm{Elastic}\ \mathrm{unloading}\ \mathrm{of}\ \mathrm{inelastic}\ \mathrm{contact}\kern1em \mathrm{f}={\mathrm{k}}_{\mathrm{e}}\left(\upalpha -{\upalpha}_{\mathrm{p}}\right) $$

5 $$ \mathrm{Overcoming}\ \mathrm{adhesion}\ \mathrm{beyond}\ \mathrm{detachment}\kern1.75em \mathrm{f}=-{\mathrm{k}}_{\mathrm{e}}\left(\upalpha -2\cdotp {\upalpha}_{\mathrm{cp}}+{\upalpha}_{\mathrm{p}}\right) $$

(ii) Viscoelastoplastic adhesive according to Morrissey [55] and Thakur [56]

The model follows the adhesive interactions according to the JKR theory [51] and is piecewise non-linear— by raising the stiffnesses k in the piecewise linear model of Luding [52] using an exponent n. The model also includes a realistic prediction of the damping force f_d_ evaluated from the velocity-dependent coefficient of restitution e. The key expressions are presented in Eqs. (6-9).

6 $$ \mathrm{Viscoelastoplastic}\ \mathrm{loading}\kern1em \mathrm{f}={\mathrm{f}}_0+\left[\left(4/3\right)\cdotp \surd \mathrm{R}\ast \cdotp \mathrm{E}\ast \right)\cdotp {\upalpha}_{\mathrm{n}}\Big]+{\mathrm{f}}_{\mathrm{d}} $$

7 $$ \mathrm{Viscoelastic}\ \mathrm{unloading}\kern0.5em \mathrm{f}={\mathrm{k}}_{\mathrm{p}}\left({\upalpha}_{\mathrm{n}}-{\upalpha}_{\mathrm{p}}^{\mathrm{n}}\right)+{\mathrm{f}}_{\mathrm{d}} $$

8 $$ \mathrm{Damping}\ \mathrm{force}\kern1.25em {\mathrm{f}}_{\mathrm{d}}=\Big[-2\cdot \left[\surd \left(5/6\right)\right]\cdot \mathrm{\ss}\cdot \left[\surd \left({\mathrm{k}}_{\mathrm{e}}\cdot {\mathrm{m}}^{\ast}\right)\cdot \mathrm{v}\right]\operatorname{} $$

9 $$ \mathrm{Damping}\ \mathrm{coefficient}\kern1em \mathrm{\ss}=\left[\ln\ \mathrm{e}/\left(\mathrm{l}{\mathrm{n}}_2\ \mathrm{e}+{\pi}_2\right)\right] $$

The symbols m’ and v represent the effective mass of the contacts, and the solid velocity of the contact, respectively.

(iii) Further considerations

**Fig. 7:**
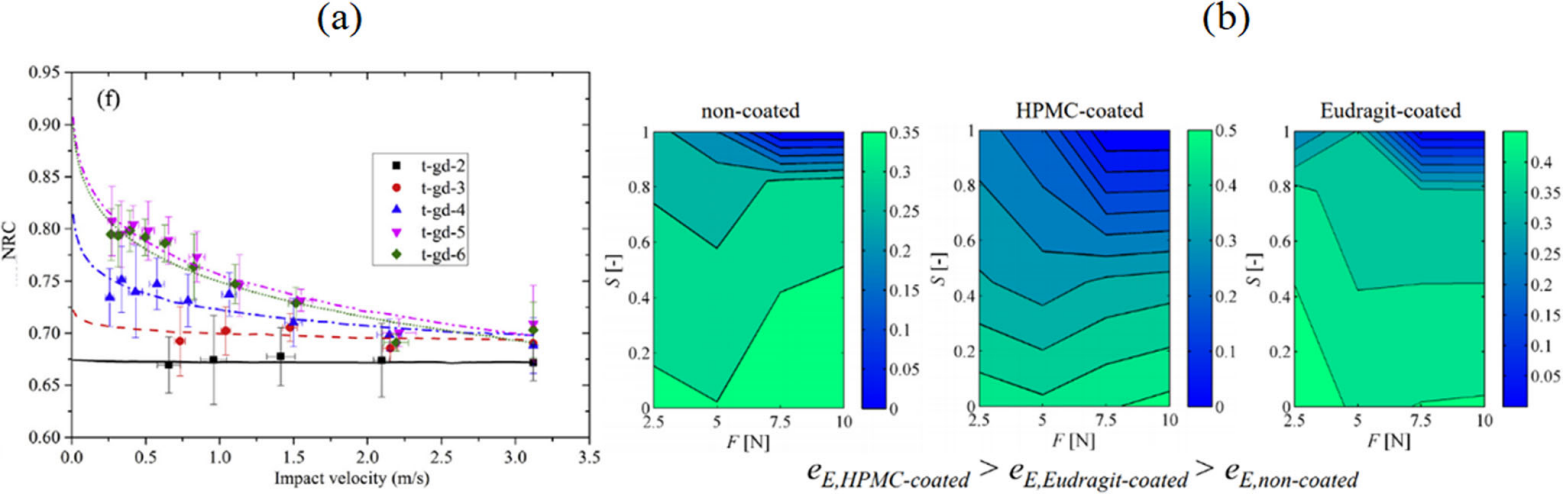
Experimentally measured restitution coefficients **a** marble articles of sizes 2–6 mm; reproduced from 59, **b** MCC pellets (d_50_ = 1 mm) with 20-μm-thick HPMC and Eudragit based coatings, containing different moisture contents (represented here by pore saturation degree ‘S’) and stressed at different contact forces; reproduced from [60]

Often the challenge in accurately representing the regimes of deformation where elasticity and plasticity alternate in governing the total deformation is the unknown pressure-dependent transition points, especially when strain hardening occurs in the material. Brake [57] proposed an elastoplastic contact model that incorporates the limits of elasticity and plasticity represented by two mutually complementing transition functions, supplemented with strain hardening and interfacial tension for low contact forces. This has been supplemented to consider plastic effects during restitution by Big-Alabo et al. [58]. Overall, the approach constitutes a deeper analysis when significant deformation can occur in powder processing, especially for materials with low elastic tolerances. Although the restitution coefficient is chosen as a constant parameter for the entirety of a process, experimental studies have shown that it may be significantly changing during a process, depending on the collision velocities and evolving particle surface properties, e.g., size, moisture content (Fig. 7). The viscoelastic size-dependent model proposed by Ye and Zhang [59] promises the benefit of optimizing DEM simulations of processes where size reduction and increase may occur. Such models that include the evolution of particle properties may serve as the next generation of accurate predictive modeling.

Models such as the ones mentioned in this section provide a detailed description of the contact interactions, thus making the modeled system as representative as possible to the real-world case. Nevertheless, this is done at the expense of introducing a large number of parameters that are laborious to measure or estimate. The computational model parameters are then either estimated based on measured data on a particle or contact level or by measuring the bulk behavior and calibrating the model parameters until the same bulk response is achieved. The ideal calibration procedure choice may be unique for the components, aim, and details of the case being studied. Coetzee [61] reviewed the existing calibration approaches towards the goal of developing standardized and validated approaches for consideration of targeted physical attributes.

#### Future Outlook for Flow Modeling

Continuum models simplify the flow modeling and are being proven beneficial, see [62] for instance; especially since there is limited agreement on rheological models for granular flow. For blends where segregation could occur, coupling rheology with process-inherent segregation modes is essential [63]. Monte Carlo methods consider only the final states and therefore transition mechanisms are neglected. FEM, DEM, and DEM-CFD although computationally expensive, help significantly in understanding particle behavior in secluded timepoints that can be critical to altering the state of the bulk powder.

A thorough risk-based evaluation of which models represent material dynamics and kinematics with sufficient accuracy for robust process control of each unit operation (sub-process) can be immensely beneficial when standardized and built-in to development platforms. Studies to demonstrate the limitations and advantages of the integration of such unit-operation-specific models to holistically represent a manufacturing process are desirable. This can significantly help in understanding process limits, such that robust control strategies can be defined to eliminate fluctuations that can potentially impact product quality and manufacturability. Furthermore, continuous data-based optimization of these models can help to leverage cross-product knowledge (e.g., potential risk for segregation of comparably cohesive powders with similar compressibility and permeability, processed in a specific equipment train).

### Powder Blending

Blending of dry powders is likely the most ubiquitous of all operations in solid dosage unit pharmaceutical processing. While simple in concept — the transformation of a powder blend from a bulk segregated state of its components to a randomly dispersed state — the quantitative prediction of the in-process state of mixedness of a blend of components based on first principles seems insurmountable without introducing simplifying assumptions and uncertainty. Multiple physical mechanisms may be at work simultaneously, including particle-particle forces (e.g., due to applied stress, adhesion and friction), as well as dispersion, convection, and shearing at the macro scale. Yet, understanding the blending operation is essential since it directly impacts the content uniformity of active pharmaceutical ingredient (API), which is one of the drug product’s most critical quality attributes [64, 65]. In addition, blending a lubricant into a formulation to enable tablet compaction in a rotary tablet press may represent another challenge. Here, maximizing uniformity is not the goal and in fact may be detrimental to achieving other desired drug product quality attributes (i.e., tablet robustness and drug release). Instead, minimally sufficient dispersion to maintain a steady film of lubricant on punch and die surfaces is the objective. Despite the seemingly intractable nature of this inherently stochastic process, some predictive methods have been developed and applied.

#### Quantifying Mixedness

If the inputs to a selected predictive method are the material properties and/or the process parameters, the output of the model will be a metric representing the state of mixedness of the blend, or perhaps a calculated value based on that estimate. The theoretical endpoint of a blending operation of discrete particles is a disordered state corresponding to ideal random mixing (IRM), whereas the initial state is often a configuration of layers of blend components sequentially charged to the blending device. From a configurational entropy point of view, achieving the greatest disorder is the goal for maximizing uniformity in powder blending. It, therefore, seems natural that the entropy approach can be applied to quantify the extent of mixedness [66, 67]. If a binary blend of particles *A* and *B* can be visualized as a 2-D grid, then the entropy *s* of each grid element *k* can be defined analogously to Boltzmann’s formula as shown in Eq. (10), where *x* is the number fraction of particles in the grid.

10 $$ {s}_k={x}_k^{(A)}\ln {x}_k^{(A)}+{x}_k^{(B)}\ln {x}_k^{(B)} $$

An overall Mixed Entropy (ME) index can then be calculated for the *N* grid elements, where *n* is the number of particles in the grid element [68]:

11 $$ \mathrm{ME}=\frac{1}{N}\sum \limits_{k=1}^N{s}_k\ {n}_k $$

Apparent from this entropy analysis is that the mixing index is dependent on how fine is the grid that is being applied. In other words, the size of the sample extracted from the blender, or the “scale of scrutiny”, directly influences the calculation of uniformity and indeed this is true for any variance-based mixing index [69]. Among this type of mixing index the most commonly used in pharmaceutical manufacturing is Relative Standard Deviation (RSD), or Coefficient of Variation *C*_*v*_. This can be readily calculated from component concentrations obtained directly from analytical testing (see Eq. (12)). Here *x* refers to the measured potency of one of *N* sample volumes extracted from the blend.

12 $$ {C}_{v, IRM}=\frac{\sqrt{\frac{1}{N-1}\sum \limits_{i=1}^N{\left({x}_i-\overline{x}\right)}^2}}{\overline{x}} $$

#### Predicting the Ideal Random Mixing Limit

If the goal of a blending operation is to approach IRM as closely as possible, estimating the degree of mixedness at IRM is a reasonable first step since it represents the achievable limit of uniformity. By definition, the probability associated with any one particle in a sampled volume being an API particle (as opposed to a non-API particle) is the same as its proportion in the mixture in the ideally random mixed state [70]. Thus, binomial statistics can be applied to assess the mean and standard deviation of API concentration within the sampled volume. Utilizing the equations for the mean and standard deviation of a binomial distribution, together with the assumptions of a monodisperse powder of spherical particles of homogenous density, the Coefficient of Variation of a single component (i.e., API) of samples extracted from a blend concentration is given by Eq. (13):

13 $$ {C}_{v, IRM}=\sqrt{\frac{\pi \rho}{6M}{d}^3\frac{\left(1-p\right)}{p}} $$

In this equation, *M* is the mass of the sample, *ρ* is particle density, *d* is particle diameter, and *p* is the probability of a particle being API. However, monodispersed particles are not a realistic assumption for actual pharmaceutical powders, and this equation can only provide rough guidance based on a d_50_ for a certain particle size distribution. Johnson [71, 72] provided a substantially improved estimate for polydisperse powders based on Stange’s [73] and Poole’s [74] earlier work, where a particle size distribution of API was divided into size classes of characteristic diameter *d* and mass fraction *f*:

14 $$ {C}_{v, IRM}=\sqrt{\frac{\pi \rho}{6M}\sum \limits_{i=1}^N{f}_i{d}_i^3} $$

Yakowlsky and Bolton [75] adapted this result for a specific distribution to the more general case of lognormal distributions typical of powders and applied this formalism to estimating the probability of passing the USP content uniformity criteria at that time. Egermann et al [76–78] have employed percolation theory to predict content uniformity for binary mixtures. Rohrs et al. [79] updated the Yakowsky and Bolton equation to use distribution statistical descriptors more appropriate for lognormal distributions, resulting in Eq. (15):

15 $$ {C}_{v, IRM}=\sqrt{\frac{\pi \rho}{6M}\sum \limits_{i=1}^N\left({e}^{3\mu +13.5{\sigma}^2}\right)} $$

While closed-form statistical approaches such as the above exist, computational models have been used to model the stochastic process of particle-level sampling from a randomly mixed blend of varying properties. Zhang and Johnson [80] and Johnson [81] proposed deterministic models for sampling from a particle size distribution to assess content uniformity. Huang and Ku [82] offered a true Monte Carlo approach utilizing Poisson sampling across all particle size fractions, demonstrating that lognormal distributions showing skew can yield significant deviations from the normality assumption. (Note: the previously mentioned studies do not specifically address granular formulations where excluded volume of granules can complicate the IRM assumption and complicate the inclusion of granular drug loading in the uniformity calculation. See Rane et al. [83].)

#### Predicting Mixedness in Process

Prediction of how quickly the mixedness of a powder blend approaches IRM minimally involves the calculation of a mixing rate, which researchers have approached using several modeling strategies.

##### Empirical Models

The solution of the diffusion equation results in an exponential relation [84], and since dispersion is a macro-scale analog of diffusion, it is not surprising that an exponential decay model of the form in Eq. (16) has been successfully fitted to dispersion-dominated axial mixing [85]. Here, *N* is the number of revolutions, *A* is a pre-exponential constant and *λ* is a rate constant. Yet, even in mixers where convection is also playing a large role, the same exponential form has been shown to approximate the mixing rate [86, 87], although the rate constants in convective mixing are orders of magnitude greater than dispersive mixing [88]

16 $$ {C}_v(N)=A{e}^{-\lambda N} $$

Nakamura et al [89] showed that their experimentally observed mixing rate was strongly correlated with an empirical powder flowability index consisting of four individual flow tests. Kushner and co-workers [90, 91] adapted the exponential decay model to the blending of magnesium stearate in V-blenders, bin blenders, and lab-scale turbula blenders to assess the detrimental impact of dispersion of lubricant on tablet tensile strength. By virtue of a scale-invariant transformation, this approach can be useful for maintaining the extent of lubrication and resulting in compressed tablet robustness upon scale-up. Lou et al. [92] modified the Kushner equation by including a lubricant cohesivity term and reported a better model fit.

##### Semi-empirical Models

Dimensional analysis is commonly classified as a semi-empirical modeling approach used in scaling where correlations are found between algebraic functions of dimensionless groups. More commonly, the simplified approach of keeping a dimensionless group itself constant, when applicable, is employed in practice to achieve scale-up. The Froude number, a dimensionless group characterizing the ratio of flow inertia to gravitational inertia, has been suggested for this purpose [93], although experimental verification for its applicability in achieving a constant mixing rate is lacking. In a rotating system the Froude number may be expressed as in Eq. (17), where Ω is rotation rate, *R* is the characteristic radius of the blending device, and *g* is the gravitational acceleration. The scaled ratio of rotation rates between two blenders is therefore predicted by the Froude number to be a weak function of their respective characteristic dimensions.

17 $$ \mathrm{Fr}=\frac{\Omega^2R}{g} $$

Alexander and Muzzio [94] have proposed an alternative approach under the tacit assumption that a dimensionless particle velocity may correlate to a mixing rate constant, arriving at this scaling equation:

18 $$ \frac{v}{k}=R{\Omega}^{2/3}{\left(\frac{g}{d}\right)}^{1/6} $$

In Eq. (18), *v* is the particle velocity in the cascading region of the bed and *k* is a fitted constant with the same dimensions as velocity. However, this approach was offered as a tentative means of achieving constant scaling without experimental verification, and further, particle velocity is unlikely to be the only factor to be considered regarding the mixing rate. For example, cohesive forces which may be playing a strong role in small blenders would seem to be less influential when scaled up to larger blenders, by virtue of surface area-volume scaling [95].

##### Continuum/Constitutive Models

Constitutive approaches for predicting powder flowability have been commonplace since the pioneering work of Jenike [29, 30]. Blending of powders via analytical flow field prediction has also been modeled, often employing numerous simplifying assumptions and limited to at most two-dimensional problems to remain tractable [96–99]. For one-dimensional systems (i.e., simple rotating drums), the dispersion model shown in Eq. (19) (also called the diffusion-convection model) has been applied to investigate simple systems such as rotating drum mixers [100]. The model describes the changes in concentration as a function of the location and the time due to the convective transport of particle collectives and random particle motions.

19 $$ \frac{\partial c\left(\xi, t\Big)\right)}{\partial t}=\Delta \left[c\left(\xi, t\right)\ D\left(\xi, t\right)\right]-\Delta \left[c\left(\xi, t\right)\ U\left(\xi, t\right)\right] $$

where *c* is concentration, *ξ* is a spatial coordinate, *t* is time and *D* is the dispersion coefficient. Recently, Liu and co-workers [101] developed a three-dimensional constitutive model combining a constitutive advection-diffusion equation with FEM to generate simulated transient macroscopic velocity fields using experimentally obtained particle dispersion correlations at a local scale. The model was applied to a three-dimensional tote blender to obtain mixing rate predictions which quantitatively compared well to published experimental data.

##### Discrete Models (DEM)

Discrete Element Modeling (DEM) is a computational technique used for simulating particle level dynamics within a powder system based on Newton’s laws of motion. Theoretical particle-particle contact models govern the powder dynamics arising from particle collisions. The enormous number of particles in an actual system precludes the direct use of DEM for simulating a blending unit operation with pharmaceutical powders. More typically, DEM is instead employed to develop insight into the effects of equipment and processing parameters such as blender rotation speed, particle size, particle shape, and initial loading patterns, and baffle design on mixing dynamics [66, 102–107].

##### Stochastic Models

The Monte Carlo method uses statistical rules to calculate the probability of the motion of particles and is relatively simple to implement as well as computationally efficient. Monte Carlo techniques have been used to study several aspects of granular behavior. Simulation of purely diffusional mixing has been performed using this technique [108], but it is more commonly used for predicting segregation in batch systems [109, 110].

#### Future Outlook for Blend Modeling

In comparison with process development of other pharmaceutical unit operations, batch blending tends to be overlooked regarding the application of modeling and simulation. The reason for this may simply be that blending is not a highly parameterized process. Sufficient process performance can often be achieved by applying just a few rules of thumb (e.g., not overloading the blender) and then increasing the number of blender revolutions until a consistent level of mixedness is measured. Batch blending, for this reason, seems to be a unit operation generally relegated to the application of standard empirical methods for development and scale-up instead.

Yet, what is the recourse when the rules of thumb do fail in process development? When material properties and blender design conspire to complicate scale-up late in development, predictive methods have the potential for saving significant cost and time even for batch blending. Hybrid modeling approaches appear to present a promising path forward. As mentioned, Yiu et al. [99] recently combined FEM with an advection-diffusion multi-scale model, yielding a predictive tool for estimating the rate of mixing that is also intrinsically scaleable since it is not particle-size based. DEM will always be limited to some extent due to a large number of particles in a typical blender, but DEM can elucidate the impact of particle properties or process parameters and in doing so may provide inputs to other models or predictive equations. Combination approaches may prove to be a productive area of exploration for blend modeling and simulation. Another recent hybrid approach combined DEM with Population Balance Modeling to hurdle the computational limitations of pure DEM for powder mixing modeling [111]. By incorporating the mixing dynamics as predicted by DEM with PBM in tumbling and agitated blender designs, the blending performance of the entire system was extrapolated from a limited DEM simulation. These types of hybrid modeling approaches may accelerate the predictive capability of simulations for powder blending in the near future.

### Tableting

The compaction of powder materials is of critical importance to a wide range of industrial applications. Examples of where the compaction process proves to be instrumental are in the powder metallurgy [112] and ceramics industries [113, 114] where powders are typically compacted to high relative densities before being sintered to produce high strength engineering components. Other industries include the food industry [115], but most notable is the pharmaceutical industry where powders are pressed to high relative densities without sintering to create solid oral dosage forms as final products (i.e., tablets). Pharmaceutical tablets easily represent the most dominant form of drug delivery utilized in the administration of medications, making up approximately 80% of all medicinal therapies [116]. Owing in part to the tablet’s dominance as a delivery vehicle for therapeutic medications is the simplicity of the die compaction process and the ability to produce a large volume of tablets consisting of various shapes, sizes, and active pharmaceutical ingredients (API).

In the development of oral solid dosage forms pertaining to pharmaceutical tablets, a pharmaceutical scientist must consider the many aspects affecting the tablet performance, such as the tablet shape and the combination of the APIs and excipients that will yield tablets with adequate strength and bioavailability. The properties of compressed powders are affected by the properties making up the powder assembly and the physics of interactions between particle-particle and particle-tooling surfaces taking place before, during, and after compaction. For example, frictional effects between the powder and powder tooling can produce inhomogeneous distributions of density and residual stress, which may result in regions of low density that are associated with inadequate mechanical strength [117–119]. This inadequate strength may be due to the properties of the material at a given solid fraction or it may be the result of the formation of microcracks.

In post-compaction processes, environmental influences can also affect tablet integrity. For example, in one study pertaining to the effect of moisture on bilayer tablet integrity [120], it was shown that layer interface decohesion was due to the differential expansion of the two layers from ambient moisture uptake. Other examples include the effect of excipient concentration, such as the effect of lubrication on mechanical strength [121] or the effect of particle size on mechanical strength.

#### Understanding Tablet Strength

While the compaction of powders offers an attractive and effective means of creating a vehicle for drug delivery, there are often difficulties associated with achieving tablets with acceptable mechanical properties. As such, drug product development pertaining to tableting has evolved from more of a trial-and-error-based approach to a science and physics-based approach that considers the chemical properties, the material science-related properties, and mechanical properties of the powder blend. The ability to predict the manufacturability and mechanical performance of a given powder blend greatly accelerates the development of formulation drug products. In pursuit of the ability to better predict outcomes in tableting design, practitioners of pharmaceutical science have turned to try to understand the various phenomena responsible for the evolution of tablet strength and tablet failure.

The strength of compacted powder materials has been examined extensively throughout the fields of soil science, powder science, and pharmaceutical science. The connection between the physics of the interactions between particle surfaces and the associated generation of bonding surface areas during the compaction of powders has been recognized as the primary prerequisites of compact strength [51, 122–124]. The formation of a solid compact from powder materials can be subdivided into several mechanisms. Examples of mechanisms that in some way influence particle-particle bonding include particle rearrangement, elastic deformation, plastic deformation, and possibly fragmentation. In the context of particle-particle bonding in the compaction process, the word “bond” refers to the formation of interfacial (contact) areas because of the well-known van der Waals interactions. For compressed powders that have undergone sufficient loading, the bonding between particle surfaces results in the creation of a solid compact with a certain degree of mechanical strength. During the compression stage of the compaction process, particle-particle bonding contact areas reach a maximum at the end of the compression where the compaction pressure is highest; however, these contact areas may be reduced once the pressure is removed. If there is a sufficient degree of stored elastic energy capable of overcoming the work of adhesion at the contact interfaces, the result will be an increase in volume, a decrease in relative density, and the formation of defects. Consequently, the formation of these defects results in a reduction of the material stiffness and degradation of cohesion in the compact. This material degradation refers to damage; a process characterized by the development, growth, and coalescence of microcracks. There is the possibility for these microcracks to coalesce to form macrocracks. When macrocracks are produced, the full separation of layers may follow, giving rise to the familiar capping and lamination failures.

**Fig. 8:**
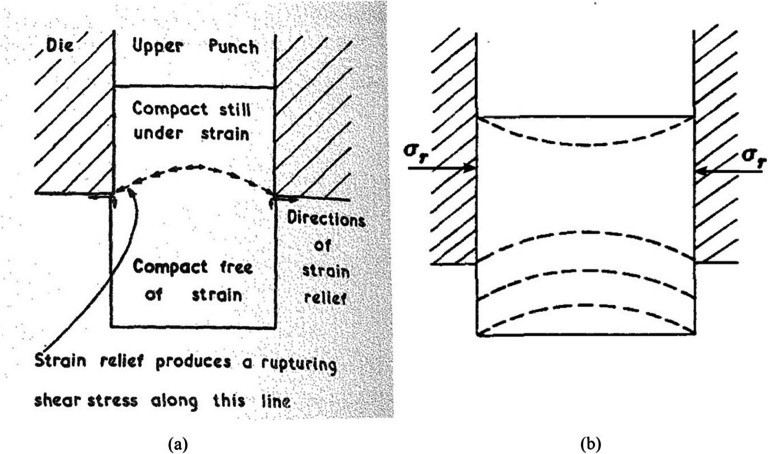
Schematic representations of lamination in **a** powder compacts suggested by Train (image adapted from [125]) and **b** capping and lamination failures as suggested by Long (image adapted from [126]). Radial stress is given by σ_r_

**Fig. 9:**
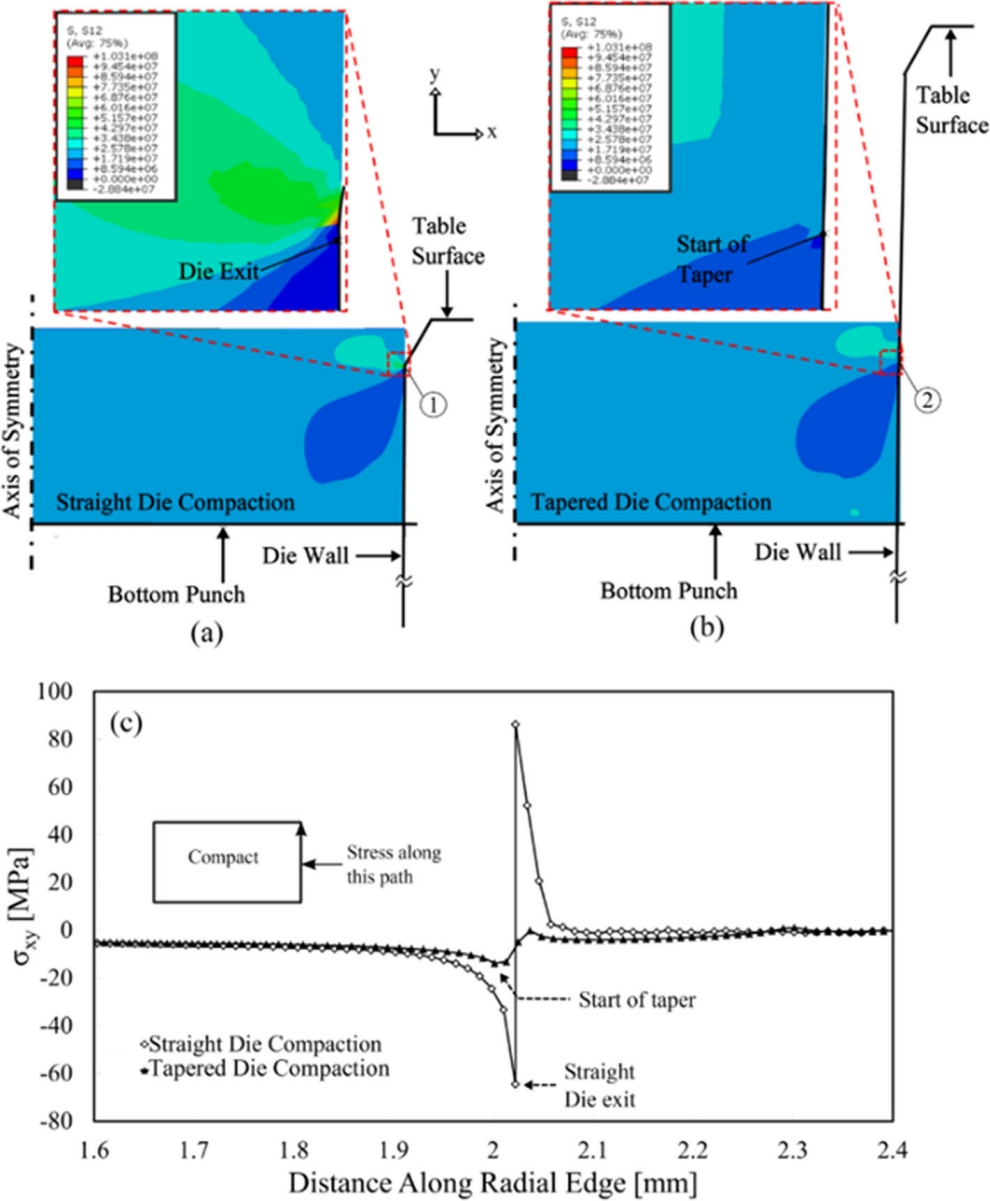
Contours of shear stress within the compacts during partial ejection from a straight **a** and a tapered die **b**. The graph in **c** shows the variation of the shear stresses along the outer edge of the compact. σ_xy_ is shear stress (figure reproduced from [127])

Research has proposed several possible explanations for the observed failures in tablets. Train [125] suggested that the development of a laminar crack through the material was the “spontaneous expansion” in both the axial and radial directions of the material exiting the die during the ejection stage (see Figure 8(a)). While Train was able to recognize that lamination failures were in some way related to the expansion of the material from the die exit, the idea that expansion alone is responsible for damage and lamination failure is not entirely correct. Capping and lamination failures of compacted cylindrical tablets were also examined by Long [126] who proposed that this type of failure was the result of a “combination of the axial expansion of the tablet exiting the die and residual die-wall pressure” (see Fig. 9(b)), another concept that is not entirely correct but it did highlight the importance of the residual die wall pressure and the die exit as a potential source for compact failures.

More recent research has focused on the origin and evolution of damage during the compaction unloading/ejection cycles to describe the mechanisms pertaining to tablet strength and tablet damage by utilizing straight and tapered dies. Garner et al. [127] utilized experimental and numerical approaches to show that diffuse microcracking develops in compacted powders upon the removal of the axial load within the die. Upon removal of the axial load, the triaxial stress state changes from high triaxiality at the end of loading to low triaxiality at full unloading. In the high triaxiality stress state, any pore or defect is essentially closed. However, as the low triaxiality stress state is approached, the residual radial wall stresses begin to dominate. Garner et al. showed that the reduction in the residual wall stress during unloading was achieved by the work that was spent in the creation of diffuse microcracks throughout the tablet, and these diffuse microcracks grow under the action of elastic expansion and shear stresses in mode II along the surface of the tablet during exiting the die. They were able to show that the utilization of a tapered die significantly reduces the detrimental effect of crack growth at the die exit by minimizing the level of shear stresses developed.

#### Powder Compaction Modeling and Simulation

Defects in powder compacts can arise from a variety of factors including any of the unit operations in the compaction process, and the quality of the raw materials in terms of their flow, compressibility, and ejection properties. The cost of defects and compact failures for manufacturers of pressed powder parts results in lost revenue, lost time, and decreased productivity. While a technician with considerable experience and training can often identify and resolve issues related to defects in the pressing of compacts, the intrinsic complexity that exists between multiple parameters and interactions requires a better understanding of the physics that underlies the process to avoid the possibility of being confronted with issues from the start. As such, researchers and practitioners of pharmaceutical science have utilized various modeling techniques to try and not only predict the mechanical behavioral outcomes, but also to understand the underlying physics responsible for the evolution of strength and damage in the die compaction process for pharmaceutical tablets. Two modeling approaches are generally utilized for this purpose and are categorized into continuum and discrete modeling techniques.

##### Continuum Mechanics Modeling Approach

**Fig. 10:**
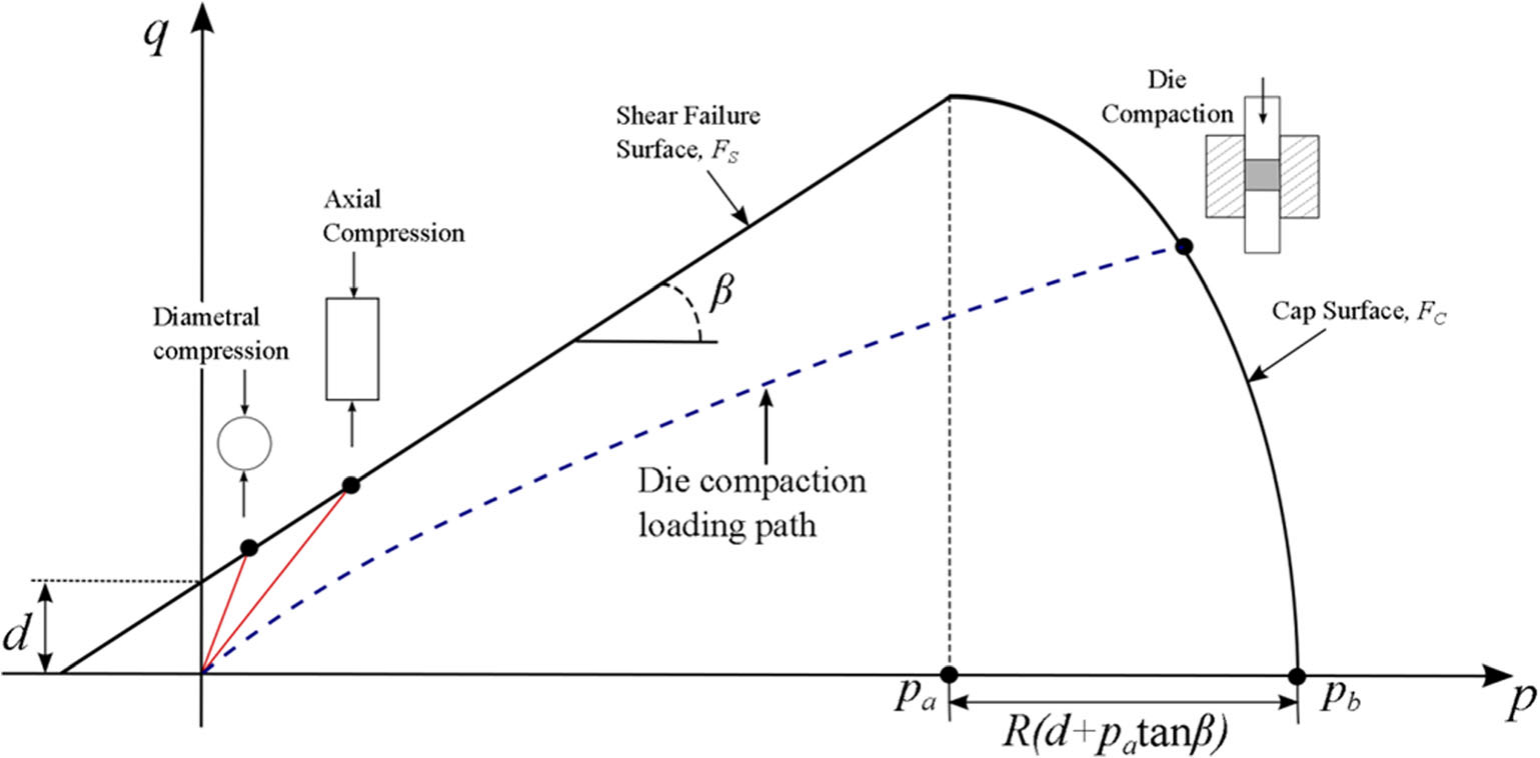
Modified Drucker–Prager/Cap model: yield surface in p–q plane with experimental procedures for determining the shear failure surface F_s_ and the cap surface F_c_. [Image adapted from [134]]

**Fig. 11:**
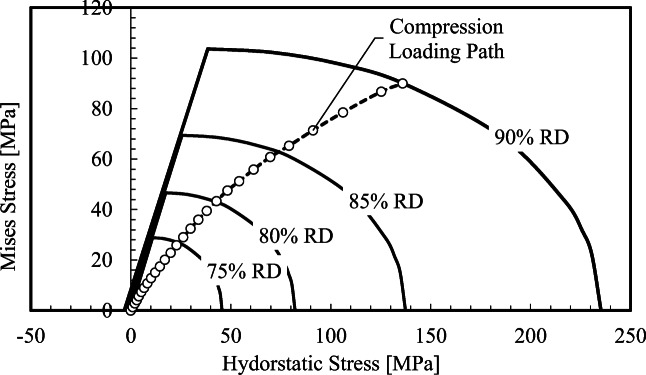
Example family of yield loci for various relative densities

Finite element-based continuum mechanics modeling is a common tool used for predicting the behavior of granular material under compressive loads [117, 128–131]. Arguably, the most accepted continuum-based phenomenological model for simulating the compaction of pharmaceutical powders is the Drucker-Prager/Cap (DPC) plasticity model [132]. Owing to its popularity is the ability to calibrate the model from a small number of experiments. The DPC model provides an inelastic hardening mechanism that accounts for plastic deformation during compaction and volume dilatancy when the material yields in shear. Central to this model is the yield surface (shown in Figure 10) which is divided into two principal segments: a shear failure surface *F*_*s*_ that describes the behavior of the powder under low hydrostatic pressure, and a cap surface *F*_*c*_ that describes hardening behavior and densification of the powder. This model is typically defined in terms of the *p-q* plane in which *p* is the hydrostatic stress and *q* is the deviatoric or Mises stress. In the *p-q* plane, the shear failure surface is represented as a straight line and is defined by

20 $$ {F}_s=q-d-p\tan \left(\beta \right)=0 $$

where *p* is the hydrostatic stress, *q* is the Von-Mises effective stress, *b* is the failure line angle, and *d* is the cohesion. The cap yield surface describing the densification of the powder is an ellipse given by

21 $$ {F}_c=\sqrt{{\left(p-{p}_a\right)}^2+{(Rq)}^2}-R\left(d+{p}_a\tan \beta \right)=0 $$

where R is a measure of the eccentricity or shape of the ellipse, and p_a_ is the point along the p-axis that represents the intersection of the shear and cap surfaces and is termed the evolution parameter. As the material densifies the yield surface shown in Figure 10 expands and the evolution of this expanding yield surface is described by the hardening law p_b_, known as the hydrostatic yield stress, as a function of the volumetric plastic strain${\varepsilon}_V^p$. Figure 11 shows an expanding yield surface at various and increasing out-of-die relative densities (RD). The five material parameters d, β, R and p_a_, and p_b_ are functions of the out-of-die relative density. For an in-depth account of the calibration procedure for the DPC model, refer to [117, 133, 134].

The use of the DPC model in the continuum model framework attempts to phenomenologically describe the kinematics and mechanical behavior of powder materials modeled as a continuum rather than as discrete particles. As such, this framework describes failure based on a continuum description dictated by the stress state corresponding to the failure surface *F*_*s*_*.* Therefore, the relative movements, the orientation of contacting particles, and evolution of coordination number *Z*, the number of nearest neighbors in contact with the particle of interest, were not considered in this framework. Such considerations have been shown to affect the mechanical behavior of compacting powders [135–138] in the micromechanical analysis of contacting particles during compaction. Furthermore, the realization that the strength of compacted powder materials is related to not only the level of interparticle cohesion but also the discrete nature of damage or fracture at the contacts means that some fundamental aspects of strength as it relates to the formation of defects are missed using continuum modeling [139]. Due to the inability of continuum approaches to capture the complexity of the particulate nature of powder materials, discrete models have emerged as a way to assess the mechanical response of contacting particles, which will be discussed in the next section.

##### Discrete Element Modeling of Compaction

The discrete element method has been widely covered in the literature by many researchers since its inception by Cundall and Strack in 1979 [140]. A comprehensive review in terms of the general understanding of the discrete element method has been established by O’Sullivan [141]. Another review on the theoretical developments, major applications, and findings of DEM was produced by Zhu et al. [142, 143]. DEM approach offers a much-improved method for understanding the physical phenomena of the compaction of powders at the microscale over continuum modeling approaches. Currently, DEM is the only available technique that can provide insight into the problem of failure at the particle level that is practical computationally. Interesting possibilities are also offered by the Multi-Particle Finite Element Method (MPFEM), see [144, 145]. However, the added computational complexity restricts the use of the MPFEM to 2-D [146] or 3-D problems with a small number of particles [147].

**Fig. 12:**
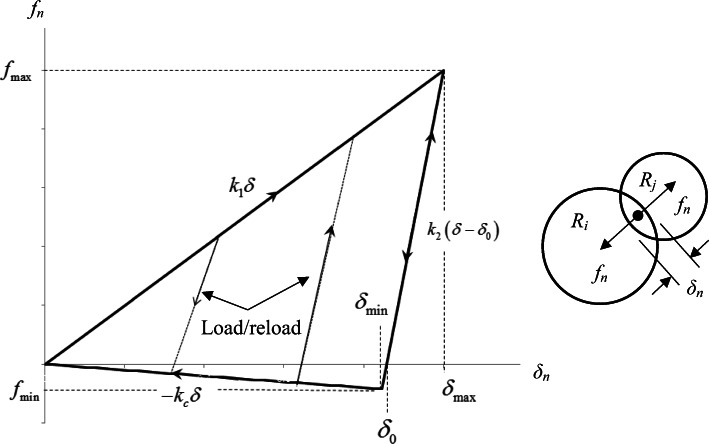
Schematic of the force-displacement contact model developed by Luding for two particles in contact

While most DEM work in the literature is usually relegated to powder flow problems, there has been significant progress made utilizing DEM for powder compaction—specifically die compaction. Prior efforts regarding die compaction using DEM can be separated into several categories: (a) simulations at conditions that do not represent typical compaction pressure and relative densities [148–150]; (b) simulations that possess force-displacement laws that take into account the plastic deformation but in approximate ways and lack consideration for contact interactions at high densities [151–154]; (c) simulations that use reasonably complex models to present the underlying physics in compression [155–160]; and (d) simulations that utilize heuristic normal contact models that approximate the physical phenomena of particle-particle loading, unloading, tension, and bond breakage often referred to as elastoplastic cohesive models [50, 161–165]. Perhaps the most well-known and most often implemented elastoplastic cohesive normal contact model in DEM is the model put forth by Luding [162]. Luding has introduced a model to obtain the relevant macroscopic mechanical behavior of granular assemblies under various loading conditions. This model is a four-parameter model (*k*_*1*_, ${\mathrm{k}}_2^{\mathrm{max}}$, *k*_*c*_, and δ^∗^) that is defined by three distinct linear springs stiffness for contacting pairs, where a spring stiffness *k*_*1*_ is the stiffness representing the loading phase of the contact, *k*_*2*_ is the stiffness representing the unloading of the contact pairs, and *k*_*c*_ is the stiffness representing the cohesive stiffness. From these stiffness parameters, the piece-wise defined force as a function of overlap is given by

22 $$ {f}_n=\left\{\begin{array}{c}{k}_1{\delta}_n\kern1.5em \mathrm{if}\ {k}_2\left({\delta}_n-{\delta}_0\right)\ge {k}_1{\delta}_n\\ {}{k}_2\left({\delta}_n-{\delta}_0\right)\ \mathrm{if}\ {k}_1{\delta}_n>{k}_2\left({\delta}_n-{\delta}_0\right)>-\\ {}-{k}_c{\delta}_n\kern2em \mathrm{if}-{k}_c{\delta}_n\ge {k}_2\left({\delta}_n-{\delta}_0\right)\end{array}\right.{k}_c{\delta}_n $$

where *δ*_*0*_ is defined as the plastic contact deformation. Figure 12 shows a schematic representation of the Luding model for normal contact between two spheres.

The Luding model is considered adequate for lightly compressed powders subjected to low-stress triaxiality stress conditions; however, the same cannot be said for powder materials subjected to much larger, high-stress triaxialities most often encountered in pharmaceutical tableting. The pressure-density curve in Figure 12 increases linearly over the entire relative interparticle strain, which for highly confined particles in the die compaction process is not correct. Furthermore, the particle-particle cohesion does not generally show the gradual decrease in bond strength for particles that have undergone large plastic deformation as is depicted in Figure 12, but rather, the bond strength decreases rapidly as Mesarovic and Johnson have indicated [124]. Both theory and experimental work demonstrated that as an RD = 1 is approached, the pressure-density relationship exhibits an exponential or power-law increase in pressure [134, 166–168].

**Fig. 13:**
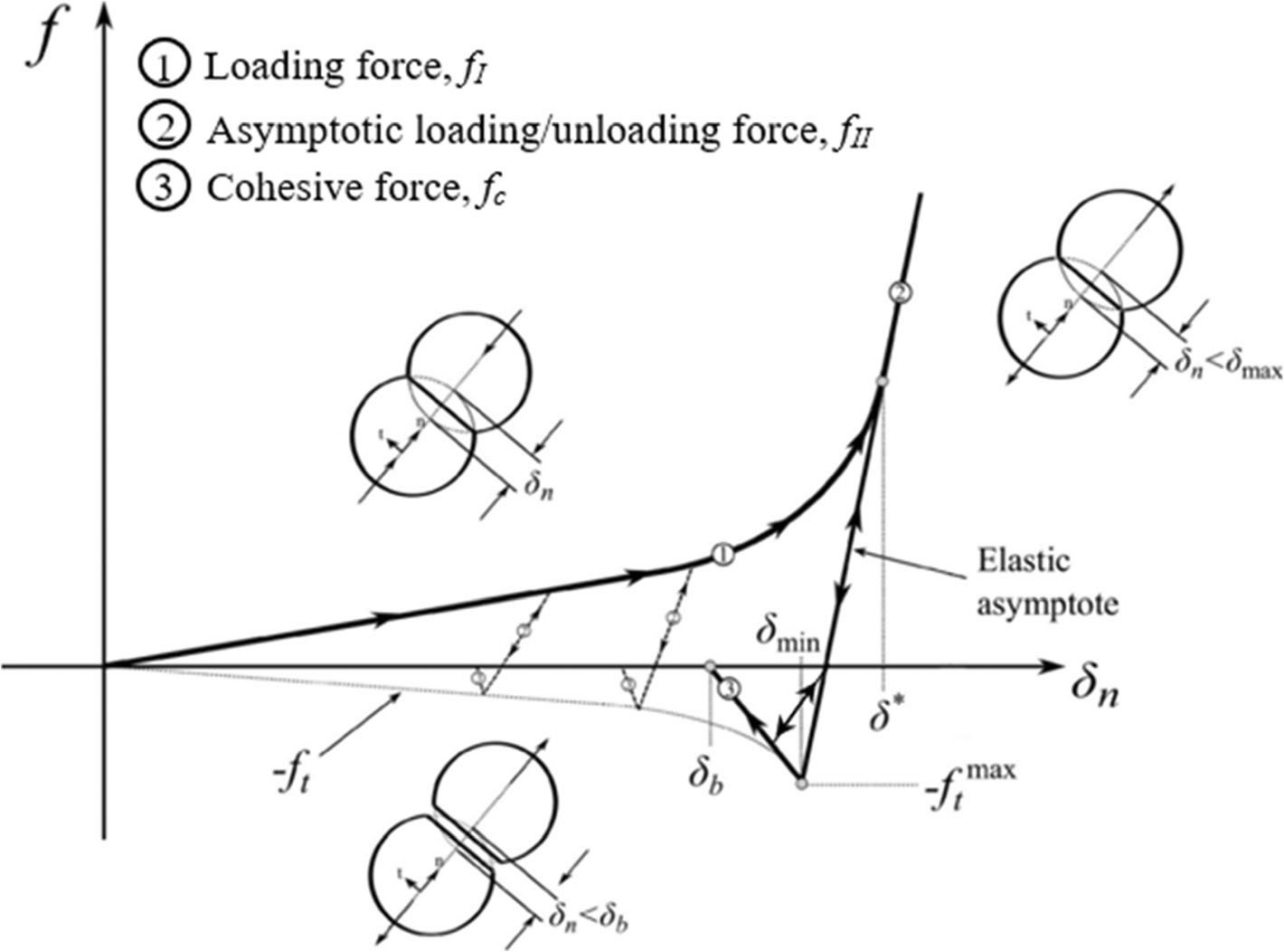
Schematic diagram of the normal force-displacement behavior in the proposed contact model

To address this discrepancy, researchers have improved on the deficiencies of the Luding model by incorporating the physical phenomena necessary to describe the loading, unloading, and cohesion for particles subjected to large plastic deformation and high confinement [163, 164]. Garner et al. [164] developed an adhesive elastoplastic contact model that describes the force-displacement behavior of contacting particles subjected to high confining conditions. The formulation of the proposed contact model took a heuristic approach to model the pressing of powders to high density. Although the approach was an approximation to the real behavior of contacting particles subjected to high confinement, the model demonstrated the ability to predict and capture the behavior of powders in the compaction process. Figure 13 shows the proposed normal force-displacement behavior of particle contacts subjected to high confinement. This model was developed similarly to the model proposed by Luding but included terms describing the upturn of the force-displacement law as the pores close, and a transition to a linear asymptotic force-displacement occurs due to the particle becoming nearly incompressible in the absence of plasticity. This transition occurs when a critical strain is reached. To represent cohesion that develops due to plastic deformation on a contact, a simple bilinear model of the response under tension was used. This representation of the cohesive interaction between particle contacts was a simplification of the Mesorvic and Johnson model. A maximum tensile pull-off force *f*_*t*_ that can be withstood by the contact was described by Garner et al. to be proportional to the maximum compressive force *f*_max_ that the contact was previously subjected to at the end of loading. This is an assumption that remains to be verified experimentally in the future. After the maximum tensile force was reached, a rapid decrease in the load-carrying capacity of contacts was assumed.

Unlike the well-established DPC model used in the continuum description of granular material, calibration of contact model parameters for DEM is not straightforward. A sensitivity analysis for each of the parameters in a particular contact model is usually the first approach. The ideal outcome of a parameter sensitivity analysis should allow for the calibration of the model parameters to match DEM results for a real material system. Using this approach, it would be necessary to establish the range for which each of the model parameters can vary. In some cases, it is not possible to determine the exact range of values, and some sound judgment must be used to determine the range of values for each of the parameters used in a proposed DEM contact model. For example, it is quite difficult to know, a priori, the range of possible values of a parameter (e.g., *k*_*1*_*, k*_*2*_*,* and *k*_*c*_ in Eq. (22)) that will lead to a desirable response that closely matches experimental results without rigorous experimental testing. It is therefore important to also establish the nature of experimental testing that will be used for calibration. The two possibilities for experimental testing are tests performed at the local level or tests performed on macroscopic samples. Tests at the local level may include, for example, atomic force microscopy (AFM) using colloidal probes (i.e., colloidal probe microscopy) to obtain results about the cohesive properties of the contacts. While it has been shown that this method of obtaining information about cohesive properties is possible, this type of testing can be tedious, time-consuming, and impractical for establishing the cohesive behavior of particle-particle contacts in DEM contact models. Examples include large variability in the results (e.g., [169, 170]), as well as the fact the typical colloidal probes are on the order of hundreds of nanometers (e.g., [171]), which are much smaller than typical particle sizes used in powder compaction applications. It is felt that results obtained from using these small probes are not representative of the real particle system typically handled in pharmaceuticals.

In general, performing tests on individual particles is difficult, to say the least. Therefore, testing on macroscopic samples utilizing statistical techniques tends to be a more practical and prudent approach. A method that has been used extensively for the screening of parameters in experimental studies is the *Design of Experiments*(DOE) procedure. The DOE procedure is an effective method for planning experiments to obtain data for analysis of multiple variables that affect a response efficiently and systematically. This method has also found wide use in the extraction of parameters of micro-mechanical models and has proved to be a successful method for the determination of optimal model parameters. One of the first works to apply the DOE method to DEM models was performed by Yoon [172] to calibrate bonded particle (i.e., non-penetrating, hard spheres) contact model parameters for simulations involving the uniaxial compression of 2-D circular disks with elastic material properties representative of rock materials. Favier et al. [173] used DOE methods to calibrate DEM models for mixing and hopper-based simulations. More recently, a rather robust work related to the use of DOE for the calibration of contact model parameters was introduced in the Ph.D. thesis by Johnstone [174], in which contact model parameters were calibrated for contact models related to flow. Specific DOE methods, such as the Taguchi DOE method, have been used for the calibration of bonded agglomerates by Hanley et al. [175]. A more recent work conducted by Garner et al. [164] utilized a combined DOE-optimization approach by first performing a central-composite-design(CCD) DOE to generate different responses of elastic and plastic energy per unit volume as a function of varying DEM model contact parameters, and then optimizing for those parameters to match the evolution of axial and radial stresses in loading and unloading for a real particle system of Copovidone-basedhot-melt-extruded(HME) amorphous solid dispersion powder.

#### Future Outlook for Compaction Modeling

While the modeling of the powder compaction process has advanced substantially over the past decades, there are still many unanswered questions about the complex behavior of compressed powders and the ability to sufficiently model these complex behaviors mechanistically. There has been great interest in the ability to quantify, predict, and mitigate tablet damage in the compaction and post compaction processes. To that end, some recent developments in the fracture mechanics space have focused on the use of specialized modeling techniques to predict fracture in powder materials implemented in FEM. An example of one such specialized technique has been put forth by Paluzny et al. [176] in which a C++-based, three-dimensional FEM simulator for fracture growth and fragmentation of granular materials has been used to examine channelized flow in geomechanically generated discrete fracture networks. This method has been termed the Imperial College Geomechanics Toolkit (ICGT) and it is capable of naturally predicting the evolution of fracture and damage without the need to specify fracture locations within a FEM mesh—a necessity for currently available modeling techniques implemented in FEM, such as the extended finite element method (XFEM) or cohesive zone modeling using cohesive finite elements. This unique approach could potentially form the ability to predict at an early stage the manufacturability (e.g., tabletability) and performance of a given blend, and greatly facilitate the robust and efficient development of formulation drug products.

A recent specialized FEM technique used to examine the strength and damage of compacted powder was conducted by Loidolt et al. [177] in which the multi-particle finite element method (MPFEM) popularized by Gethin et al. [144] and Procopio et al. [145] was updated to include cohesive contact between FEM particle boundaries. In addition, a novel way of incorporating periodic boundary conditions was introduced, which allowed for the generation of an efficient representative volume element (RVE) for compaction of powder and the description of yield surfaces as a function of different cohesive particle-particle contact strengths. With further work to investigate the flow rule of the material upon yielding and the elastic constitutive behavior of the particle system by utilizing this MPFEM approach, it may be possible to inform the implementation of a continuum model with the ability to simulate the macroscopic compression of an entire powder system in the compaction process.

**Fig. 14:**
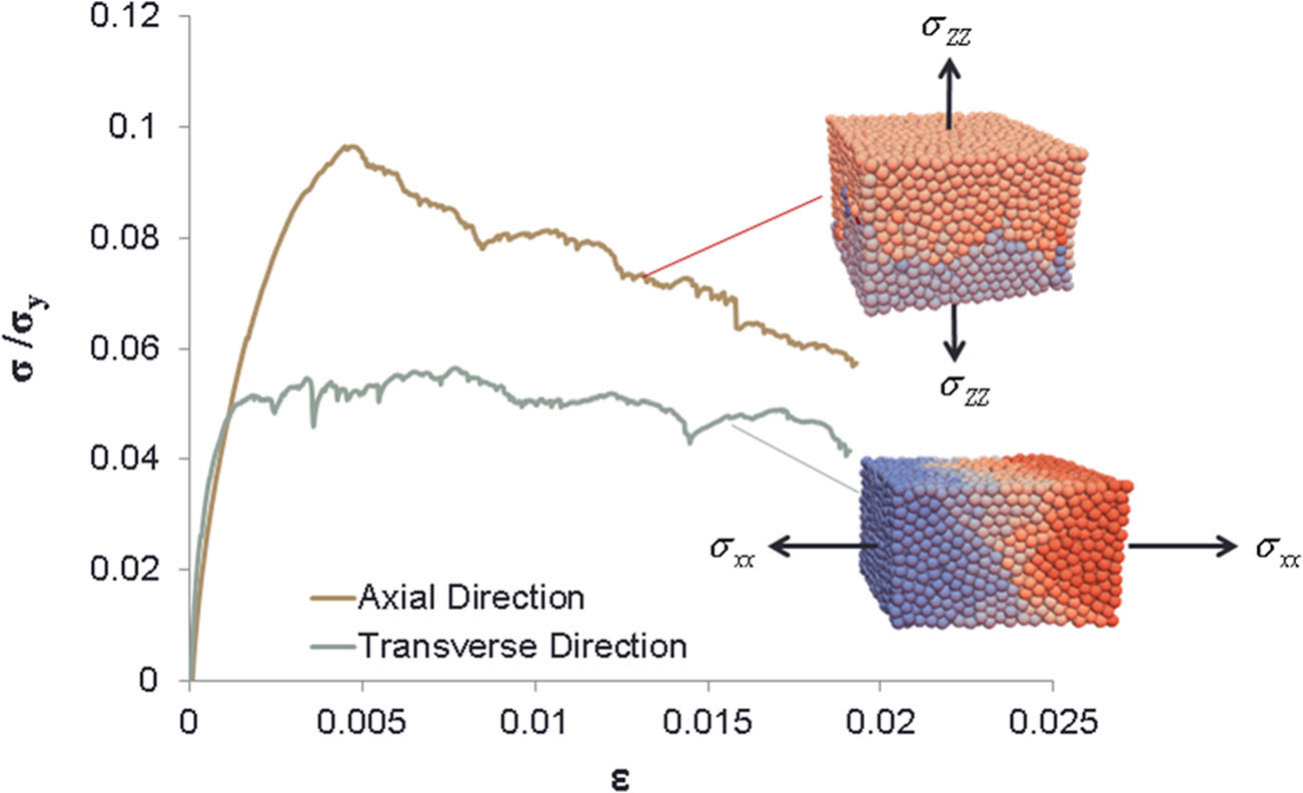
Incorrect prediction of strength anisotropy for transversely confined and uniaxially compressed powders (figure reproduced from [178])

Unlike FEM, DEM has not found substantial widespread use within pharmaceutical industrial settings for modeling and simulating the tableting process. While DEM work presented in Section 2.3.2.2 shows some key advantages over other numerical techniques in terms of DEM’s ability to naturally account for the discontinuous nature of particle-particle contact separation, there remain some critical aspects of DEM that need to be addressed for powder compaction modeling. For the vast majority of DEM implementations, the typical contact models implemented do not incorporate contact-contact interactions but instead assume each contact as being independent of one another. Although this assumption is adequate for small interparticle strains such as in problems related to particle flow, it is not a proper assumption for the large interparticle strains and stresses experienced by particles in the powder compaction process. A possible unfortunate consequence of excluding contact-contact interactions is the improper prediction of strength anisotropy as shown in Figure 14.

Models developed by Gonzalez et al. [160], Tsigginos et al. [157], and Frenning [155, 156] have attempted to address the contact-contact interactions by incorporating the dependence of forces acting elsewhere on a particle; however, employing these models in a typical DEM implementation may require a fundamental change to the way contact forces are calculated, thereby increasing computational complexity and computational time. Because of the added complexity and computation time, the implementation of these contact-contact interaction models may currently be viewed as impractical for use in simulations involving a large number of particles; however, with the advent of GPU computing and the ever present push toward increased computational power, it is likely that these models will be more practical to use in the future.

### Tablet Film Coating

Film coats are applied to tablets for a range of aesthetic or functional purposes. Such coatings can serve to mask the taste, color, or odor of the dosage form, provide additional chemical or physical protection, improve elegance, and aid in dosage form identification. Further, film coatings are also used to modify the API release profile or can provide a means to apply an active pharmaceutical ingredient onto the tablet surface.

Historically, batch film coating processes utilizing rotating film coating pans have been typically used to coat pharmaceutical tablets. More recently, continuous processing has received greater attention throughout the industry and, consequently, some continuous tablet coating equipment has become commercially available. In addition, other film coating processes, for example, Wurster coating processes [179, 180], are used to coat mini-tablets. Despite differences in the equipment geometry and some other specifics that may vary somewhat, each of these film coating processes relies on the same key physical phenomena to produce quality tablet coatings.

**Fig. 15:**
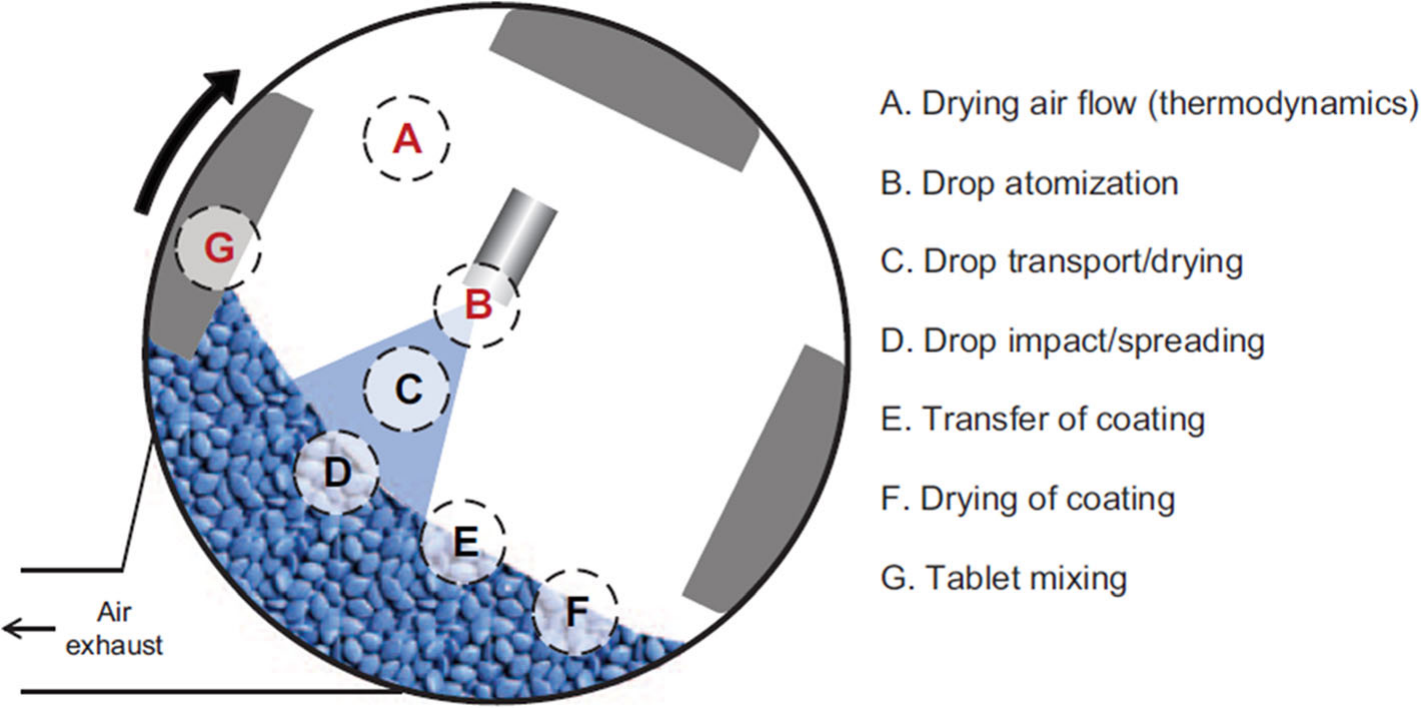
A schematic of the tablet coating process highlighting several key physical phenomena. Reproduced from Ketterhagen et al. [181] with permission

A successful film coating process relies on the success of several interrelated physical phenomena. These key phenomena are illustrated in a schematic of a coating process shown in Fig. 15. The flow of warm, dry air through the tablet bed controls the drying process within the pan of the coating process (A). The coating fluid is pumped through one or more spray nozzles to be atomized (B) and then flows toward the tablet bed below (C). Upon reaching the tablet bed, the drops will tend to impact the tablet cores, wet the tablet surface, and begin spreading over the tablet cores to create the film (D). Many other phenomena may also be occurring including spray drying, the rebound of droplets off tablets, and absorption of water into the tablet cores. The spreading coating material may also transfer to neighboring tablets (E) before drying into a film on the tablet surface (F). While these processes continue for the duration of the coating process, tablet mixing (G) due to the pan rotation and the mixing action of the internal baffles helps to ensure good coating uniformity is obtained. The success of each of these physical phenomena is critical to the success of the coating process as a whole. Should there be issues with one or more of these phenomena, coating quality issues such as twinning, picking/sticking, surface roughness, undesired equilibrium water content, or coating mass non-uniformity may arise.

The aforementioned physical phenomena can be further classified into macro-scale phenomena and micro-scale phenomena following Turton [182]. The macro-scale phenomena encompass those processes occurring throughout the coating pan and affect the entire batch of tablets. In contrast, micro-scale phenomena occur at a local, tablet surface scale and include processes such as drop impact, spreading, liquid absorption, and evaporation. Prediction of these micro-scale phenomena becomes important to capture finer level details on individual tablets (e.g., film morphology and surface roughness) that cannot be adequately described by macro-scale phenomena models alone.

A few of the macro-scale physical phenomena — namely coating pan thermodynamics, drop atomization, and tablet mixing — have been well-studied throughout the past few decades and have been summarized in recent reviews [181–183]. Due to this previous coverage, these models are only briefly described here. In contrast, the micro-scale phenomena have been far less studied with respect to pharmaceutical coating operations until recent years.

#### Macro-Scale Phenomena

Numerous thermodynamic modeling approaches have been developed and applied to tablet film coating processes over the past few decades [184, 185] and have been the topic of recent reviews [181]. These approaches typically rely on mass and energy balances over a control volume encompassing the coating pan and may include a heat loss factor describing heat loss to the environment. The process can also be represented graphically on a psychrometric chart [186]. The models have been applied to systems involving either aqueous or organic coatings and, in either case, describe the environmental conditions in the coating pan in terms of an environmental equivalency (EE) or as a temperature and relative humidity of the outlet airflow. These approaches can be particularly useful during process transfer or scale-up activities, where they can be used to ensure environmental similarity across varying process equipment.

The atomization of the film coating fluid into fine droplets is another process where significant modeling attention has been devoted [187]. Various analytical expressions for mean drop size, often described by the Sauter mean diameter (SMD), have been proposed. The work by Varga et al. [188] and Aliseda et al. [189] consider the breakup of the liquid jet through a sequential process of a primary Kelvin–Helmholtz instability that disrupts the liquid jet and a secondary Rayleigh–Taylor instability that breaks the cylinder into droplets. These researchers have proposed expressions for the SMD that are largely governed by a dimensionless mass flow ratio, and the Weber, Reynolds, and Ohnesorge numbers. These analytical models are useful during process transfer and scale-up activities to estimate typical spray drop size and ensure similarity across equipment that may utilize different spray nozzles. Later work by Niblett et al. [190] extended the Aliseda et al. [191] model to include an empirical factor describing the coalescence of droplets during the time of flight toward the tablet bed. This factor was shown to markedly increase droplet size for some conditions.

The uniformity of film coating is an important quality attribute of the coating process that can be described by the inter-tablet coating variability (i.e., the variability in coating mass across all tablets within a batch) and the intra-tablet coating variability (i.e., the variability of coating across the surface of a given tablet). Several modeling approaches including DEM [192–197], Monte Carlo approach [198, 199], population balance models [200, 201], and renewal theory [202, 203] have been employed to predict coating uniformity. Further, various researchers have proposed hybrid approaches making use of combinations of the aforementioned methods (e.g., Freireich et al. [204]). Each of these approaches and their relative advantages has been recently reviewed by Ketterhagen et al. [181]. Application of these coating uniformity models can provide a better understanding of how process parameters (e.g., batch size, pan speed), material properties (e.g., tablet size and shape), and equipment design (e.g., pan size, mixing baffle configuration) can impact the degree of coating variability. Often, these approaches can be used to design or transfer a coating process such that acceptable coating uniformity is achieved [192–197]. Monte Carlo approach [198, 199], population balance models [200, 201], and renewal theory [202, 203] have been employed to predict coating uniformity. Further, various researchers have proposed hybrid approaches making use of combinations of the aforementioned methods (e.g., Freireich et al. [204]). Each of these approaches and their relative advantages has been recently reviewed by Ketterhagen et al. [181]. Application of these coating uniformity models can provide a better understanding of how process parameters (e.g., batch size, pan speed), material properties (e.g., tablet size and shape), and equipment design (e.g., pan size, mixing baffle configuration) can impact the degree of coating variability. Often, these approaches can be used to design or transfer a coating process such that acceptable coating uniformity is achieved.

Predictive approaches for each of these macro-scale phenomena have proven useful in aiding the design, scale-up, and transfer of industrial film coating processes. However, each of these approaches typically requires assumptions that limit somewhat the respective predictive capabilities. These assumptions center around the fact that the local environment near the tablet is typically not considered. For example, the thermodynamic models typically make predictions of the outlet air temperature and humidity. These are correlated with, but may not exactly predict, the environmental conditions a certain tablet may experience as it passes through the spray zone and down the cascading tablet bed. Similarly, the mean droplet size can be predicted, but other important aspects such as the distribution of droplet sizes, the changes in the size distribution due to coalescence and/or drying, and the spatial variation of spray flux are more difficult to predict. Finally, the coating uniformity models typically neglect aspects of thermodynamics and the spray. Often these approaches rely on the assumption that tablet residence in the spray zone is equivalent to coating mass gain. While the models for macro-scale phenomena have been proven to be effective, further inclusion of micro-scale phenomena is anticipated to bring a greater resolution to film coating modeling predictions through more detailed predictions of coating uniformity as well as new predictions on coating appearance including surface roughness and morphology.

#### Micro-Scale Phenomena

In recent years, increasing research has focused on the development of predictive approaches for micro-scale phenomena such as drop impact on tablet cores, spreading and film formation, water absorption and evaporation, and the transfer of liquid coating between tablet cores. While many of these phenomena have been studied for long periods of time in other industries, more recent research has focused on highly viscous and complex rheology fluids more typical of film coating suspensions.

Higher-resolution spray atomization models have been developed, often arising from the automotive industry, using a variety of computational approaches such as smoothed particle hydrodynamics and volume of fluid methods (e.g., [205–207]) that provide detailed information on quantities such as drop size distribution, sphericity, and spatial distribution of droplet concentration.

The impact of droplets has been described in the experimental work of Mundo et al. [208] that revealed the Reynolds and Ohnesorge numbers govern the post-impact behavior and delineate whether the droplet will splash or deposit on the substrate. This relation has been used in subsequent modeling work, e.g., [209] to specify droplet post-impact behavior. Suzzi et al. [209] conducted multi-phase CFD simulations using the Lagrangian discrete droplet method for spray droplet modeling. The simulations investigated droplet impingement and film formation on a tablet core. Effects of film evaporation and heat transfer between the film tablet core and gas phase are also considered and predictions include mean and variance of the film coating thickness. Simulations were limited to the normal impact of spray droplets on the face of the tablet and only considered a single tablet in space, so effects such as oblique impact angles and liquid transfer were not considered. The modeling did show that the splashing of droplets was detrimental to establishing a smooth, uniform coating.

Bolleddula et al. [210] experimentally studied the impact of viscous droplets and did not observe splashing or rebounding for the conditions examined. They proposed relations for the dynamics of drop spreading as well as the maximum spreading diameter as a function of the Weber and Reynolds numbers. Dechelette et al. [211] developed a one-dimensional theoretical model for droplet spreading and elucidated some differences in the process between non-Newtonian and Newtonian fluids.

More recently, Christodoulou et al. [212] have developed modeling approaches for the spreading, absorption, and evaporation processes on the local scale of a single tablet. The approach employs (a)one-dimensional spreading models based on a mechanical energy balance to model the kinematic phase of liquid spreading just after impact, (b) solution of the continuity and Navier-Stokes equations for when the capillary effects are important during spreading and absorption, and (c) solution of energy conservation equations during the liquid evaporation phase. Predictions from these computational approaches compared favorably with experimental data for drop spreading [204] and liquid absorption into the tablet core [213]. Later work [214] extended the model to include predictions of the spreading rate and thickness of the liquid film.

After establishing this model incorporating several micro-scale phenomena, Chistodoulou et al. [214] probed the model responses through a sensitivity analysis. This analysis revealed that the film thickness established once a tablet is initially coated is primarily dependent on the droplet impact velocity, the liquid viscosity, and the droplet diameter. The spray mass flow rate did not impact film thickness, but of course, significantly affected coating application time. Smaller, secondary effects on coating time were observed for droplet impact velocity, the liquid viscosity, and the droplet diameter.

As the preceding paragraphs highlight, recent research has delved into developing a more complete understanding and predictive capability of the micro-scale phenomena within the tablet film coating process. These advances are significant and are greatly enhancing the nature of film coating modeling predictions. However, there exist opportunities to further develop these models. A variety of assumptions appearing in some of these micro-scale phenomena models could be further addressed to develop a more complete film coating process model. For example, some models are limited to large, millimeter-scale droplets and do not consider micron-scale droplets present in a typical coating process. Others make simplifying assumptions on the nature of the fluid rheology (e.g., inviscid, Newtonian). Further assumptions often include unform spray fluxes, orthogonal droplet-tablet impacts, non-porousand/or planar substrates, and/or simplified substrate wettability characteristics. As is apparent, many complexities are present within the micro-scale phenomena of the film coating process. These should provide fertile ground for future research activities.

#### Future Outlook for Film Coating Modeling

Recent developments in film coating modeling have tended to focus on the development of micro-scale phenomena models related to the intricacies of droplet/tablet impact, spreading, and drying. These new models, in partnership with the aforementioned macro-phenomena models, make possible more complete predictive approaches. In addition, there has been increased attention in integrating various film coating sub-models into comprehensive, multi-scale film coating models.

Niblett et al. [190] developed a phenomenological modeling framework consisting of a mixture of mechanistic and empirical models that included factors such as tablet velocity, drop atomization, coalescence, drying, spreading, and spray flux. In this approach, tablet appearance quality was well predicted in a regime map consisting of a dimensionless tablet drying time and a dimensionless spray flux. For cases where the dimensionless drying time is too large, defects such as logo bridging or over-wetting are observed. For cases with low spray fluxes, processing times became too long for industrial purposes. Cases with larger spray fluxes and smaller dimensionless drying time led to tablets with a good appearance. This work employs both macro-scale and micro-scale phenomena models to successfully predict tablet coating appearance.

In another integrated modeling approach, Boehling et al. [215] developed a coupled CFD-DEM based approach to model the tablet coating process in a semi-continuous pan coater. This approach simulated the tablet dynamics and the process thermodynamics in a coupled approach that allowed for the prediction of coating uniformity, outlet air temperature and relative humidity, and tablet moisture. For a range of process conditions, the simulation results agreed reasonably well with experimental data.

The outlook for predictive modeling of tablet coating is promising. The advancements in models for thermodynamics, drop atomization, and tablet mixing over the last few decades have created useful tools for practical film coating process design and scale-up. They have also set the foundation for more recent developments that have begun to elucidate the micro-scale phenomena of drop impact, spreading and film formation, absorption, and drying as they pertain to pharmaceutical tablet coating. In the coming years, several trends including further advancements in micro-scale model development and computational capabilities should lead to more powerful film coating models that include detailed, micro-scale sub-models of local coating environment and phenomena within a computational approach for tablet motion, drying airflow, and spray atomization. Such future integrated, multi-scale models could potentially predict many of the critical quality attributes of the film coating process including coating uniformity, elegance/appearance, water content.

## SUMMARY

This review highlights some impactful process modeling approaches that can be applied to study batch operations involved in a typical direct compaction drug product manufacturing process. The review begins by considering recent learnings related to the reliable feeding, discharge, and consistent flow of solid powder streams. Models to better interpret flow measurements, as well as iterative options to counter challenging powder behavior, are also highlighted. Following this, the three core unit operations — powder blending, powder compression, and tablet coating — have been individually reviewed. Each section begins with the core purpose of the unit operation, followed by targeted discussions on leveraging modeling capabilities to help build robust controlled processes. The discussed modeling capabilities range from empirical to mechanistic, from discrete to continuum phases, and from a deterministic to a probabilistic model of assessment. Many other process modeling options are not included here, which may be essential especially while handling materials of highly unique and challenging characteristics.

The presented content highlights how combining process engineering fundamentals with particle and powder technology can be regularly harnessed to solve technical challenges in the pharmaceutical industry — e.g., to inform the development of manufacturing processes [216] to guide scale-up [217], or to optimize processes based on manufacturing experiences [218]. While this review focused on modeling and simulation approaches available to inform process development, the intent is not to diminish the necessity of system-relevant experimental data and historical information required to apply these techniques to a specific formulation or formulation options available. Many of the references cited provide examples of measurement techniques, laboratory and process studies useful to applications with which the reader may be faced. Similarly, the models and simulation tools found in the cited material will guide the reader to create or refine their models to fit their formulation and process constraints and opportunities.
